# Supplementary material for: Evaluating the efficacy of Internet-Based Exercise programme Aimed at Treating knee Osteoarthritis (iBEAT-OA) in the community: a study protocol for a randomised controlled trial
Source: BMJ Open. 2019 Oct 28;9(10):e030564. doi: 10.1136/bmjopen-2019-030564 (PMC6830654; doi:10.1136/bmjopen-2019-030564)
Supplement: Supplementary data [file bmjopen-2019-030564supp001.pdf]

Time up and Go: The participant will start in a seated position.
The participant will stand up upon therapist's command, walk 3 meters, turn around, walk back to the chair and sits down.
The time will stop when the participant is seated.
The subject can use an assistive device. If the assistive device is used, it will be documented.
NOTE: A practice trial will be completed before the timed trial

30 seconds sit to stand. The 30-Second Chair Test is administered using a folding chair without arms, with a seat height of 17 inches (43.2 cm). The chair, with rubber tips on the legs, is placed against a wall to prevent it from moving.
The participant is seated in the middle of the chair, back straight; feet approximately shoulder width apart and placed on the floor at an angle slightly back from the knees, with one foot slightly in front of the other to help maintain balance. Arms are crossed at the wrists and held against the chest.
Demonstrate the task both slowly and quickly.
Have the participant practice a repetition or 2 before completing the test.
If a participant must use their arms to complete the test, they are scored 0.
The participant is encouraged to complete as many full stands as possible within 30 seconds.
The participant is instructed to fully sit between each stand.
While monitoring the participant's performance to ensure proper form, the tester silently counts the completion of each correct stand. The score is the total number of stands within 30 seconds (more than halfway up at the end of 30 seconds counts as a full stand). Incorrectly executed stands are not counted.
The 30-second chair stand involves recording the number of stands a person can complete in 30 seconds rather than the amount of time it takes to complete a pre-determined number of repetitions.

Muscle strength Assessment: Isokinetic testing will be done at 60 and 180 degrees of flexion and the participant will be in sitting position with hips and knees strapped to keep the position standardised. Isokinetic torque will be measured in the seated position on a Computer Sports Medicine, Inc (CSMI) HUMAC / NORM Testing and Rehabilitation System (Model 770) isokinetic dynamometer at 60 and 180° s−1 angular velocities. There will be 30 seconds rest between each testing and one-minute break between 60 and 180° s−1 angular velocities.

Ultrasound and Muscle Thickness Assessment (MTA) of Vastus Lateralis Oblique (VLO): An ultrasound will be used to image both knee joints using a Toshiba Aplio SSA-770A machine with a multi-frequency (7 – 12 Hz). This equipment belongs to the university and is CE marked. During the ultrasound scan, the maximal synovial thickness and effusion depth will be measured in millimetres using the longitudinal axis. Suprapatellar pouch, medial and lateral recess of the knees will be assessed for synovial thickening, synovial fluid/effusion and for positive power Doppler.

Maximal muscle thickness will be measured in transversal images as the distance between the superficial and the deep fascia at the widest distance and scan will be conducted at the midpoint between greater trochanter and knee joint. The pennation angle is defined as the angle between muscle fibres and the deep fascia of the muscle. Pennation angles will be measured in the longitudinal ultrasound image for three fibres of vastus medialis, and the average of these three measurements will be used for further analysis.

An ultrasonic probe will be used to direct ultrasonic waves onto the knee joint during sonography, and a computer converts the signals received so that they can be presented on the screen. There is no radiation exposure to ultrasound due to the lack of radioactive rays and no detrimental side effects.

Pressure Pain threshold (PPT)

PPT is a non-invasive test during which the sensitivities of the nerves are assessed by recording the smallest force applied to the skin. When this force by the surface area of the skin is applied (pressure), this will be felt as mild, temporary pain and recorded. The pressure probe used consists of a rod with an end the size of a 5p piece, mounted in a handheld device connected to a computer. The force with which the probe is pressed onto the skin is gradually increased until the participant indicates (by pressing a button) that the sensation has changed from pressure to pain. The probe is then automatically immediately taken off the skin. The probe will be used on knee (medial joint line, supero-lateral, supero-medial and tibialis anterior) using a standardised protocol used in other studies within the Pain Centre. The participants will be familiarised with the test before it is administered so that they know what to expect and how to respond.

Temporal Summation (TS)

The mechanical temporal summation is a non-invasive test during which repetitive mechanical stimulation is applied over a short period to get their augmented response. Increased pain response to a repeated mechanical stimulus may indicate enhanced central sensitisation. The test site will be a suprapatellar region (5 cm proximal from the central part of patella).
A 256mN weighted pinprick stimulator will be used and applied perpendicular to the skin of suprapatellar region of affected knee (5cm proximal from the centre of patella). The participant will be asked to rate the pain or sharpness they experience from 0-10 where 0 indicates no pain or sharpness and 10 indicates the most intense pain or sharpness imaginable. Numerical Rating Scale (NRS) with verbal descriptors will be used. The response of the participant will be recorded. The same stimulator at the same site will be applied ten times repeatedly at a rate of 1/second. The size of the site would be kept approximately 1 cm square. At the end of the series of 10 pinpricks, the participant will be asked to rate the pain or sharpness which they experience averaged over the whole series of 10 stimuli using the same NRS. The mechanical temporal summation reading will be calculated as the difference between two ratings which is second rating minus the first rating.

Continuous Pain Modulation (CPM)

CPM will be done along with PPT testing. The reference point for PPT testing will tibialis anterior on the most painful knee. Verbal instructions will be given to the participant. The Numerical Rating Scale (NRS) target will be ≥4 out of 10 from the cuff pressure. Staff/ clinician will wrap 7.5cm wide tourniquet cuff around the contralateral arm to the knee being tested. The lower rim of tourniquet cuff will be kept 3cm proximal to cubital fossa. Systolic pressure will be set to 20mmHg higher than systolic blood pressure of the participant. After target pressure is achieved, the participant will be asked to rate sensation in the arm from 0-10. The participant will be asked to make hand grips until NRS of 4 will be reached. NRS rating will be asked every five hand grips.
Once NRS of 4 will be achieved, the probe of algometer will be applied in the same manner as before to tibialis anterior site (during PPT testing). Once the participant presses the button, the probe will be withdrawn, and cuff will be released from the elbow. Participants will be advised to wait until cuff evoked pain subsides before re-test, and a minimum of 1 minute should be spared. PPT test will be repeated again (without the cuff now). Their difference in PPT score (with conditioning – without conditioning) will establish the CPM effects. Positive value predicts efficient and negative value predicts in-effective CPM.

Sites for QST

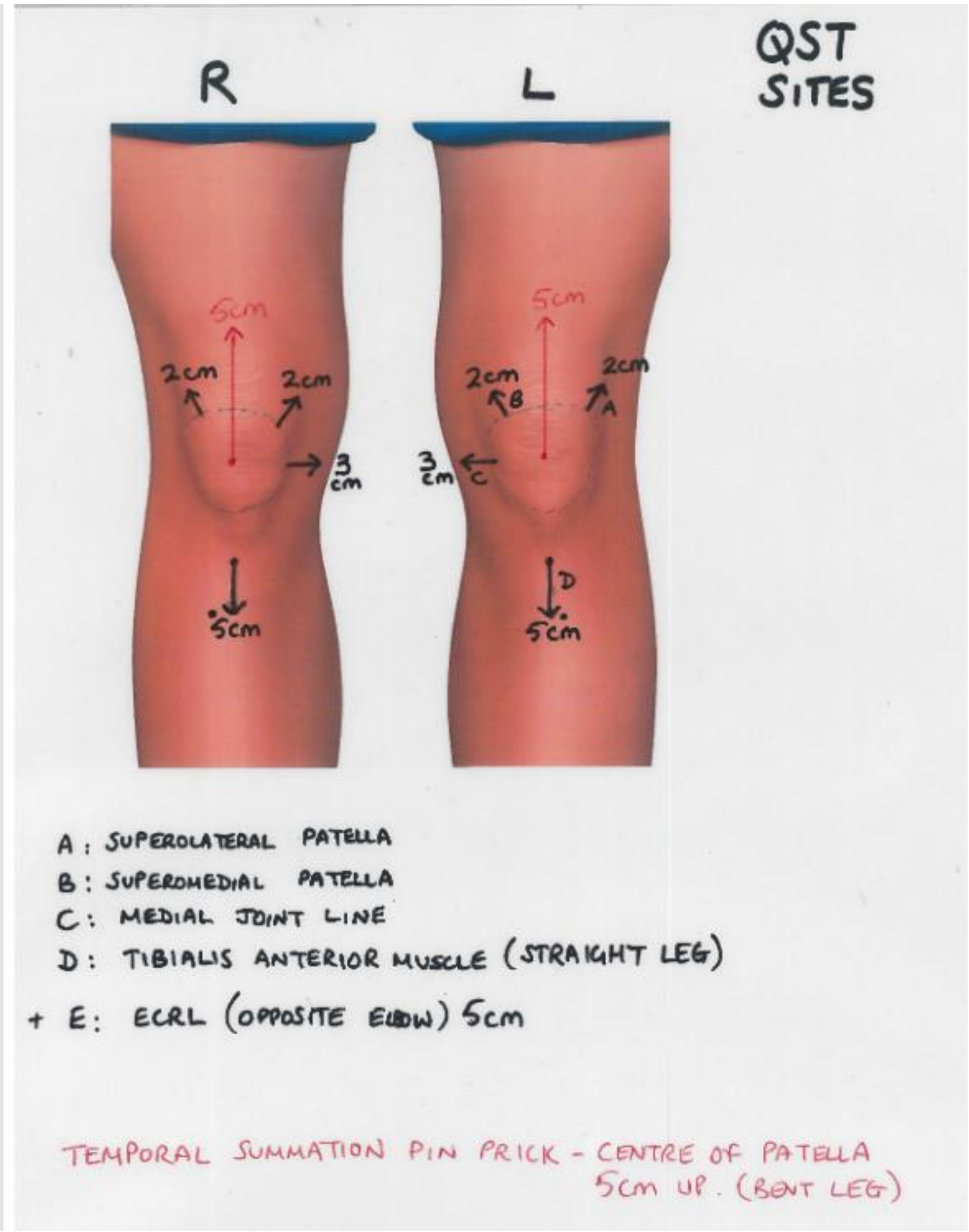

Biomarkers:
Insulin, glucose, C- reactive protein (CRP), triglycerides, LDL, HDL, TNF-alpha, IL6,
