# Supplementary material for: Evaluating the efficacy of Internet-Based Exercise programme Aimed at Treating knee Osteoarthritis (iBEAT-OA) in the community: a study protocol for a randomised controlled trial
Source: BMJ Open. 2019 Oct 28;9(10):e030564. doi: 10.1136/bmjopen-2019-030564 (PMC6830654; doi:10.1136/bmjopen-2019-030564)
Supplement: Author's manuscript [file bmjopen-2019-030564.draft_revisions.pdf]

BMJ Open

BMJ Open is committed to open peer review. As part of this commitment we make the peer review history of every article we publish publicly available.

When an article is published we post the peer reviewers' comments and the authors' responses online. We also post the versions of the paper that were used during peer review. These are the versions that the peer review comments apply to.

The versions of the paper that follow are the versions that were submitted during the peer review process. They are not the versions of record or the final published versions. They should not be cited or distributed as the published version of this manuscript.

BMJ Open is an open access journal and the full, final, typeset and author-corrected version of record of the manuscript is available on our site with no access controls, subscription charges or pay-per-view fees (http://bmjopen.bmj.com).

If you have any questions on BMJ Open's open peer review process please email info.bmjopen@bmj.com

BMJ Open

A study protocol for a randomised controlled trial evaluating the efficacy of Internet-Based Exercise program Aimed at Treating Knee Osteoarthritis (iBEAT-OA)

Journal:	*BMJ Open*
Manuscript ID	bmjopen-2019-030564
Article Type:	Protocol
Date Submitted by the Author:	20-Mar-2019
Complete List of Authors:	Gohir, Sameer; University of Nottingham Division of Academic Rheumatology,<br>Greenhaff, Paul; University of Nottingham School of Medical and Surgical Sciences, Division of Physiology, Pharmacology and Neuroscience<br>Abhishek, A; University of Nottingham Faculty of Medicine and Health Sciences, Rheumatology, Faculty of Medicine & Health Sciences<br>Valdes, A; University of Nottingham Faculty of Medicine and Health Sciences
Keywords:	knee osteoarthritis, web based intervention, iBEAT-OA, knee pain

SCHOLARONE™
Manuscripts

A study protocol for a randomised controlled trial evaluating the efficacy of Internet-Based Exercise program Aimed at Treating Knee Osteoarthritis (iBEAT-OA)

Sameer Gohir[1], Paul Greenhaff[1,2], Abhishek Abhishek[1], Ana Valdes[1]

[1]NIHR Nottingham Biomedical Research Centre, School of Medicine, University of Nottingham, [2]MRC Arthritis Research UK Centre for MSK Ageing Research, [3]School of Life Sciences, University of Nottingham

For peer review only

Abstract

Introduction

Knee osteoarthritis (OA) is the most common joint disease worldwide. As of today, there are no disease-modifying drugs, but there is consistent evidence showing that muscle strengthening exercises can substantially reduce pain and improve function in this disorder, and one very well tested physiotherapy protocol is the "Better Management of Patients with Osteoarthritis" (BOA) developed in Sweden (1). Given the high prevalence of knee OA, a cost-effective digital based approach to treat knee OA should be trialled. This study aims to explore the benefits of an Internet-Based Exercise Program Aimed at Treating knee OA (iBEAT-OA) in modulating pain, function and other health-related markers in individuals suffering with knee OA.

Methods and Analysis

A randomised controlled trial was designed to evaluate the efficacy of a web-based exercise program in a population with the knee OA compared with standard community care provided by general practitioners (GP) in the UK. We anticipate recruiting participants in to interventional (n=67) and control (n=67) groups. The interventional group will exercise for 20-30 minutes daily for six consecutive weeks whereas the control group will follow GP recommended routine care. The participants will be assessed using a Numerical Rating Scale (NRS), the Western Ontario and McMaster Universities Osteoarthritis Index (WOMAC), the Arthritis Research UK Musculoskeletal Health Questionnaire (MSK-HQ), the Pittsburgh Sleep Quality Index (PSQI), a 30-second sit to stand test (30CST), a time to up and go test (TUG), a Quantitative Sensory Testing (QST), a musculoskeletal ultrasound scan (MSK-USS), a Muscle Thickness Assessment (MTA) of vastus lateralis, quadriceps muscles force generation during an isokinetic Maximum Voluntary Contraction (MVC). Samples of urine, blood, faeces and synovial fluid will be collected to establish biomarkers associated with changes in pain and sleep patterns in individuals affected with knee OA. Standard parametric regression methods will be used for statistical analysis.

Ethics and Dissemination

Ethical approval was obtained by the Research Ethical Committee (REC) (Ref: 18/EM/0154) and the Health Research Authority (HRA) (Protocol no: 18021). The results of the trial will be submitted for publication in a peer-reviewed journal.

Strength and Limitation of this Study

This study is novel, since to our knowledge it is first to evaluate a web-based exercise intervention to improve pain in sufferers of knee OA in the United Kingdom. Another strength is that we plan to recruit only those individuals presenting with radiographic evidence of knee OA that will rule out non-OA causes of knee pain such as soft tissue injury or patellofemoral syndrome. Moreover, this study is the first study to evaluate changes in sleep disturbance due to knee OA along with an exercise intervention.

Lastly, the mechanistic aspect of this study will provide valuable information to help unravel the complicated relationship between chronic pain, sleep, body composition, biomarkers of inflammation and knee OA.

The lack of double-blind study design is typically a limitation of exercise and lifestyle intervention studies, which cannot be avoided in this study. However, the assessors of the study end-points will be blinded. Another study limitation is the recruitment of individuals from only one geographical region only.

Introduction

Knee OA is a common cause of disability globally, and is mostly managed in the primary care (2). In the United Kingdom, 10% people between the ages of 65 and 74 consult their general practitioners (GPs) for OA every year (3). In the UK, on in twenty-five, people consult their general population for knee OA annually (2).

The first line of treatment for OA involves exercise, education and weight loss if relevant (4-8). Exercise improves joint and patient centred outcomes in people with knee OA, and encourages them to cope better with the activities of daily living (9-23). In fact, strengthening knee exercises have been shown to reduce progression of radiographic changes of knee OA (24). There is a disparity of opinion regarding the effectiveness of different types of exercise for the knee OA to reduce the pain, and a combination of open and closed isotonic exercises are recommended for knee OA (25). In an attempt to manage knee OA, symptoms of knee arthritis can be exacerbated by following ineffective or unsafe exercises regimens, leading to poor prognosis and poor adherence to exercise intervention (26). Hence the need to choose the type and frequency of exercise intervention carefully.

Arthritis Research UK (ARUK) recommend knee exercise for knee pain, which it views as a generic term covering soft tissue injuries (27). An exercise regimen is normally recommended by the GPs as a part of the first line of treatment when they consult someone with arthritis-related knee pain. If exercise intervention fails to impact positively on pain perception, patients are referred for Physiotherapy. After a comprehensive assessment by physiotherapists, a variety of exercise interventions have been prescribed that fluctuate in exercise modality, intensity, and duration, but with variable success in terms of outcome. Therefore, there is a need for standardised exercise interventions to address knee OA pain, which would ideally be accessible online to maximise efficacy testing and cost-effectiveness. The exercise intervention, which we will use in this project, is part of a web-based program derived from the Supported Osteoarthritis Self-Management Programme (SOASP), which was developed in Sweden. From January 2008 until January 2017, around 75,000 patients participated in the SOASP, 2339 physiotherapists and occupational therapists were educated to deliver the Supported Osteoarthritis Self-Management Programme, and today SOASP is offered in 700 clinics all over Sweden (28). This intervention has been rated good to very good by 94% at reducing the pain related to knee OA and has an excellent safety profile and acceptability (29).

For peer review only

Because of the high prevalence of knee arthritis, strategies to deliver exercise interventions that are both efficacious and cost-effective should be prioritised. Some of the issues relating to the delivery of exercise interventions for knee OA include compliance, accessibility to clinics for people with mobility problems and the cost of delivery of such services. There are previous studies which have assessed the efficacy of home-based exercises (11, 13, 30), however, very few studies looked at web-based delivery of exercise intervention (31-35). Unfortunately, most of these studies recruited patients with knee pain, and radiographic evidence of knee OA was only assessed in a single study (33). This makes the results of these studies less specific to base a recommended treatment for knee OA. And generalising these results on the group of patients with knee OA warrants a risk of increasing their knee pain. Therefore, there is a need for an exercise programme that is specific to Knee OA and sufficiently cost-effective that individuals can perform the intervention in the home settings.

Knee OA pain is accompanied by a number of additional disturbances that influence the individual's health, which we also propose to study in conjunction with pain relief due to exercise. There is a well-known link between sleep disturbance and chronic pain, and epidemiological studies (36-38) and experimental studies (39-43) have established a link between disturbed sleep and knee OA. These studies confirmed that individuals with OA have greater sleep disturbances. Furthermore, sleep disturbance is recognised as an important factor in determining pain perception (43). A relationship between sleep disturbance and pain severity in knee OA patients is usually explored, and sleep disturbances such as shortened sleep duration and fragmented sleep (36) have been associated with increased sensitivity to pain in OA patients, and consequently with decreased quality of life (38). Therefore, studying the sleeping pattern is highly relevant if we are discussing a successful online exercise programme for knee OA.

Recent epidemiological and clinical studies have also underlined that metabolic syndrome (MetS) has the most significant impact on the initiation and severity of OA (44-48). Metabolic triggered inflammation, also known as meta-inflammation (49) can be a result of abnormalities in body composition, adipokines, cytokines, lipids, and vitamin D and has been associated with the pathogenesis of OA (47). Given body composition is influenced by a number of the variables associated with OA, e.g., systemic inflammation, sleep patterns, altered physical activity levels, and altered energy intake, it is important to study this as well. The intricate links between exercises, sleep, pain, metabolic syndrome, body composition, and OA are not fully understood. To date, the relationship between improvements in pain due to exercise for pain relief and other changes related to health parameters that have been linked to OA or chronic pain has not been explored. Specifically, the effects of exercise for knee pain relief on sleep, biomarkers of inflammation and insulin resistance is unknown. We aim to study these parameters and establish the link between exercises for knee OA and these parameters. Figure 1 has shown this in PICOT format.

Rationale

This study aims to explore the benefits of an internet-based exercise programme in patients with knee OA to establish if six weeks intervention reduces pain perception and sensitivity. Being a web-based exercises intervention, also makes it accessible and cost-effective to volunteers and physiotherapists, and will hopefully help establish a standardised intervention for knee OA that will maintain individual motivation, and compliance, thereby managing their pain. Lastly, we will endeavour to explore the complicated relationship between chronic pain, sleep, biomarkers of inflammation, body composition, and knee OA. This study is novel as to our knowledge no study has yet evaluated a web-based intervention to improve knee OA in the United Kingdom, particularly, as we plan to recruit only those individuals presenting with radiographic evidence of knee OA that will rule out non-OA caused of knee

For peer review only

pain such as soft tissue injury or patellofemoral syndrome. Therefore, this study could potentially help generate recommendations for the treatment of knee OA.

Primary Objective

To test whether an internet-based exercise intervention can reduce pain perception in knee OA.

Secondary Objective

To test whether there is a benefit of iBEAT-OA to improve sleep disturbances, reduce pain sensitisation and metabolic syndrome.

Methods and Analysis

iBEAT-OA Trial Design

The iBEAT-OA study is a randomised controlled trial in the primary care setting at Nottingham with participants identified as having knee arthritis, 1:1 randomised to web-based exercises or usual care as shown in the Figure 2.

Setting

Home based - Primary Care Setting

Patient and Public Involvement

On the patient and public involvement (PPI) representative meeting of 14/12/2015, seven volunteers suffering from chronic arthritis pain were asked about the relevance of studying sleep in the context of OA pain. They all were very supportive of studying and understanding sleep patterns. They viewed actigraphy as a good non-invasive alternative to polysomnography and saw no problem with using the device for 6 weeks.

On 17/05/2017 6 representatives from the PPI Musculoskeletal group were asked about exercise interventions and were all supportive of this. They were also asked about the extraction of synovial fluid from their joints. Five out of six said they would not have a problem with this if it was performed by a specialist using ultrasound to guide the needle, which is why this is an optional part of the protocol.

The latest versions of the participant facing documentation (PIS, consent form, invitation letter and flyer) has all been forwarded to three PPI representatives who have reviewed and commented on it.

Recruitment

A selection of eligible people for the study will be invited from existing databases (50) held at Academic Rheumatology, City Hospital Nottingham of participants with knee pain who have agreed to be contacted for future studies. The inclusion and exclusion criteria are shown in Figure 3. Any shortfall in the recruitment will be compensated by sending study leaflets to GP surgeries. The GPs will follow the inclusion and exclusion criteria. All those individuals who are suitable for the study will be sent the study information sheet. The interested participants will contact the research centre to show their interest and will be recruited.

Ethical approval was obtained by the Research Ethical Committee (REC) (Ref: 18/EM/0154) and the Health Research Authority (HRA) (Protocol no: 18021).

For peer review only

Randomisation and blinding

The eligible participants will undergo computer-generated randomisation and allocation concealment. Randomisation will be done using software "sealed envelope" (https://www.sealedenvelope.com/), an online randomisation service. Participants will be equally allocated between treatment arms with at least n=67 per arm. At this point, individuals will be alerted to which study group they have been assigned and hence blinding is not possible. Because this might affect the motivation of participants randomised into the control arm, these individuals will be offered access to the web-based exercise programme after completing the study. Blinding of exercise intervention studies is difficult; however the study examiner will be blinded to the intervention.

Sample size and justification

We are anticipating recruiting at least 67 individuals into each study arm (with an anticipated 12% drop out rate leading to 60 individuals) at the closure of the study.

A recent systematic review of 44 high-quality exercise trials for knee OA pain (3537 participants) (51) found an average effect of 12/100 VAS points corresponding to 0.49 standard deviations. A sample size of n=60 per group is necessary to achieve 75% statistical power. The estimated dropout rate for exercise interventions is 10%, therefore, a sample of 67 per arm will be recruited to achieve 75% power.

Intervention

iBEAT-OA will use a web-based exercises platform known as Joint Academy (JA) as recent pilot studies (34, 35) demonstrated promising results for this platform. This programme is based on a Swedish face to face self-management program known as 'Artrosskolan' (*The Osteoarthritis School*), which provided structured information and exercises for knee arthritis to a relevant population suffering from knee arthritis. The efficacy of it is covered elsewhere (29). The company that produced the Joint Academy platform has given consent to use their web-based platform to conduct this study.

The exercise intervention comprises a mixture of open and close chain exercise manoeuvres, a combination of concentric and eccentric exercise modalities and is focused on the overall strength of legs including the muscles around the hips and knee joints. An open kinetic chain is defined as "a combination of successively arranged joints in which the terminal segments can move freely" (52). Closed chain exercises are exercises or movements where the distal aspect of the extremity is fixed to an object that is stationary and proximal joints move (52). There are balance enhancement exercises as well. There are educational sessions integrated into the programme covering the basics of OA, its treatment, self-managing symptoms of OA and the benefits of maintaining a healthy lifestyle.

Control Group

The control group will continue with routine self-management which is offered in the community setting.

Follow up duration

Six weeks post-intervention.

Research Assessment

Following outcome measures will be assessed:

1. Sleep

For peer review only

2. Quantitate Sensory Testing – QST (Pressure pain threshold – PPT, Temporal summation – TS, and conditional pain modulation – CPM)
3. Inflammatory markers on ultrasound (Synovial fluid, synovial hypertrophy, and hypervascularity) (MSK-USS)
4. Muscle Thickness Assessment (MTA) of Vastus Lateralis Oblique (VLO) using MSK-USS
5. Maximum Voluntary Contraction (MVC)- isokinetic contraction of Quadriceps muscle
6. Biomarkers of insulin resistance
7. Physical functioning (Time up and go test, 30-second sit to stand test)
8. Western Ontario and McMaster Universities Osteoarthritis Index (WOMAC)
9. Pittsburgh Sleep Quality Index (PSQI)
10. General health questionnaire (MSK- HQ)
11. Body composition assessment by Bioimpedance Analysis (BIA)

Start and End dates

The trial is expected to start in winter 2018 – Summer 2019 and enrolment will end in the winter 2019 – Summer 2020.

Description of Intervention

Participants seeking care for OA will be informed of the study both orally and in writing. Those who qualify for the study will give signed informed consent, and the participants will be randomised to an interventional group or control group. The exception applies to those participants who have not had a knee X-rays in the previous 12 months. These individuals will be called to the hospital, and after gaining valid consent, knee radiographs will be obtained, and their X-rays will be assessed by experienced staff. Once, a definite eligibility criterion (K/L score at least 1 or above) is established in such cases; the qualifying participants will be randomised to the interventional or control group.

Interventional groups will have an assessment session with experienced staff and a Numerical Rating Scale (NRS) (53-55), the Western Ontario and McMaster Universities Osteoarthritis Index (WOMAC) (56, 57), the Arthritis Research UK Musculoskeletal Health Questionnaire (MSK-HQ)(58, 59), the Pittsburgh Sleep Quality Index (PSQI)(60-63), a 30-second sit to stand test (30CST)(64-66), a time to up and go test (TUG)(67, 68), the Quantitative Sensory Testing (QST)(69-75), Musculoskeletal ultrasound scan (MSK-USS)(76-80), a muscle Thickness Assessment (MTA) of vastus lateralis(81, 82), quadriceps muscle force generation during an isokinetic Maximum Voluntary Contraction (MVC)(83), urine and blood samples (84, 85) will be taken at baseline. Those who consent for aspiration of synovial fluid will go through an ultrasound-guided aspiration (USGA) procedure (76-79). The interventional group will be given an actigraphy device (ActTrust). Their sleeping pattern will be recorded quantitatively.

The interventional group will then receive a link via email, which will be used to log-in to the Joint Academy online portal. After log-in has been achieved, the exercise intervention will start. This will consist of a 6-week internet-based physical therapy program that will provide information, exercise, contact details of a personal physiotherapist, education about lifestyle and behavioural changes. This intervention can be accessed using a smartphone, a tablet or a computer. The programme also encourages physical activity adherence by sending email prompts on a regular basis. Initially, participants will answer an online-questionnaire covering areas such as joint pain intensity, health-related quality of life, physical function as well as performing a physical test assessing lower limb strength. This questionnaire will form a part of the online baseline assessment. The exercise

For peer review only

intervention will consist of knee and hip exercises along with some functional activities such as sit to stand and stairs climbing.

After six weeks exercise intervention, participants will fill in the same questionnaire and perform the physical tests, to enable evaluation. There are two face-to-face meetings between participants and physiotherapist/nurse, at enrolment and after six weeks. The physiotherapist will be available via asynchronous online chat or over the phone during the 6-week study period.

The control group will continue with their routine self-management which is offered in the community setup. They will be assessed on NRS, QST, WOMAC, MSK-HQ, PSQI, 30CST, TUG, MSK-USS, MTA of vastus lateralis, MVC of Quadriceps muscle, body composition, urine and blood samples at baseline. The control group will also use actigraphy to monitor sleep patterns. They will follow the routine management of knee OA recommended of the National Institute for Health and Care Excellence (NICE), which includes non-pharmacological and pharmacological management(86). They will be re-assessed after six weeks on the same outcome measures to determine if there has been an impact of self-management strategies.

Data Management

In iBEAT-OA trial, data will be collected during the first and last session. Additional, weekly pain scores will be collected via an online portal and actigraphy will be used to collect sleep patterns. Online portal and actigraphy data will be collated by trained local research staff and data entry in a relational MS Access database will be completed in a standardised fashion. The clinical research forms (CRF) from the first and last study visit will be sent to the data entry site. A central data manager performs and monitors data entry. This will include questionnaires (MSK-HQ, PSQI, WOMAC) and data from QST, 30CST, TUG, MSK-USS, MTA of vastus lateralis, body composition and MVC of the Quadriceps muscle.

Statistical Analysis

The clinical trial data will be analysed using an intention-to-treat approach. We will compare outcome measures (e.g., pain sensitivity, pain scores, sleep patterns, and inflammatory measures) between exercise and non-exercise groups controlling for baseline scores using appropriate parametric and non-parametric statistical tests. Additional observational secondary analyses (i.e., correlations between change in sleep patterns and change in pain measures) will be carried out using parametric statistics and adjusting for the relevant covariates. The SPSS package will be used for the statistical analyses.

Adverse Events

There are no serious adverse events reported with these exercises. These exercises have been trialled on seventy-five thousand patients from 2008 to 2017 with no serious adverse events (1, 29). There is a small chance of an increase in the knee, hip or back pain (51). We will monitor the pain levels of the patient on a weekly basis using an internet-based interface to monitor any increase in the knee, hip or low back pain. If the pain exacerbates to the level that the participant starts struggling with the activities of daily living, then they will be advised to stop participating in the study and will be advised to contact their GP.

Criteria for terminating the study

As the study involves only two assessments and does not involve investigational medicinal products or medical devices, and the same intervention has not given rise to any serious adverse events in over

For peer review only

70,000 participants in Sweden, it is not envisaged that circumstances will arise that require termination of this study.

Ethics and Dissemination

The study has received approval from the Research Ethics Committee (REC) (Ref: 18/EM/0154), Health Research Authority (HRA) (Protocol no: 18021) and the Nottingham University Hospitals NHS Trust Research & Innovation (R&I) department (Ref: 18RH004).

The study results will be submitted to Arthritis Research UK, regulatory authorities, and a peer-reviewed journal for publication. Also, the results will be presented at national and international conferences. Study participants will also be informed of the results if requested.

Discussion

Knee OA remains one of the most common forms of OA and affects the majority of the population in the United Kingdom. There are various forms of treatment, which are offered to the patients suffering from the knee OA. There are non-pharmacological and pharmacological management recommended by the National Institute for Health and Care Excellence (NICE). The non-pharmacological management recommends local muscles strengthening, general aerobic fitness, weight loss and using transcutaneous electrical nerve stimulation (TENS) as an adjunct to other forms of management (86). The majority of the population requires some guidance as to what exercises should they do and get referred to the Arthritis Research UK (ARUK) website for basic exercises. If those exercises fail to make much difference or if patients struggle to understand these exercises, they are referred to a local physiotherapy department in the community setup. This means that some of these patients will have to travel to local community centres or hospitals to see a physiotherapist and learn the relevant exercises.

The iBEAT-OA platform can work in between the ARUK leaflet and a referral to physiotherapy, thus cutting the cost of travelling, saving the time of the patients wasted in travelling and the time of physiotherapists to treat other patients with more complex pathologies and needs.

The majority of physiotherapists guide their patients on the type of exercises they should follow and review them a few times before the patients are recommended to self-manage OA and at this stage these patients get discharged. The compliance of patients afterward can decline as patients may stop exercising. There are various reasons for this including adjusting lifestyle to include these exercises, pain during the exercises, lack of motivation and lack of professional to monitor the progress (87-89). The i-BEAT-OA platform is designed to keep these patients motivated, and it sends them regular e-mails to remind them. Additionally, it tracks their progress which can keep participants motivated.

Social media is a powerful platform, which offers a connection between users and is a source of social interaction for a range of individuals. This can be used to promote health and to treat patients with OA (90-92). iBEAT-OA is aimed at using social media such as internet and digital application on a mobile phone to encourage patients with knee OA to self-manage their condition. This programme will educate and train them to stay 'in-control' of their knee OA which will improve the overall quality of life. This will lead to the overall psychological well-being of our population. This study will also encourage other researchers to study digital health platform, which will be the preferred way of communication and solution to health-related issues for next generations.

This study intends to establish the link between digital exercises, muscles strength, knee inflammation, sleep disturbance, pain and severity of knee OA. The intention is to check if exercising regularly can

For peer review only

reduce the pain, knee inflammation, sleep disturbances and slow down the progression of knee OA. If this complicated link can be interpreted effectively, we may find a way to reduce the progression of the knee OA.

For peer review only

Figure 1: A randomised controlled trial evaluating the efficacy of internet-Based Exercises Aimed at Treating Knee Osteoarthritis (iBEAT-OA) – PICOT Format

Research Question

- Will iBEAT-OA reduce the knee pain?

Population

- Age 45 and above, male or female and suffering from knee osteoarthritis

Intervention

- Internet bases exercises aimed at treating knee OA (iBEAT-OA)

Comparison

- Participants with knee OA and following routine management for knee OA in the community

Outcome Measures

- NRS
- Sleep
- QST
- MSK-USS
- MTA of Vastus Lateralis Oblique (VLO) using MSK-USS
- MVC of Quadriceps
- TUG
- 30CST
- WOMAC
- PSQI
- MSK- HQ
- BIA

Time

- 6 weeks

*NRS= Numerical Rating Scale, QST= Quantitative Sensory Testing, MSK USS= Musculoskeletal Ultrasound, MTA =Muscle Thickness Assessment, MVC= Maximum Voluntary Contraction- isokinetic contraction of Quadriceps muscle, TUG = Time up and go test, 30CST= 30-second sit to stand test, WOMAC= Western Ontario and McMaster Universities Osteoarthritis Index, PSQI= Pittsburgh Sleep Quality Index, MSK-HQ= Arthritis Research UK Musculoskeletal Health Questionnaire, BIA = Bioimpedance Assessment

For peer review only - http://bmjopen.bmj.com/site/about/guidelines.xhtml

Figure 2: iBEAT-OA Trial design. The flowchart summarises the design of iBEAT-OA trial.

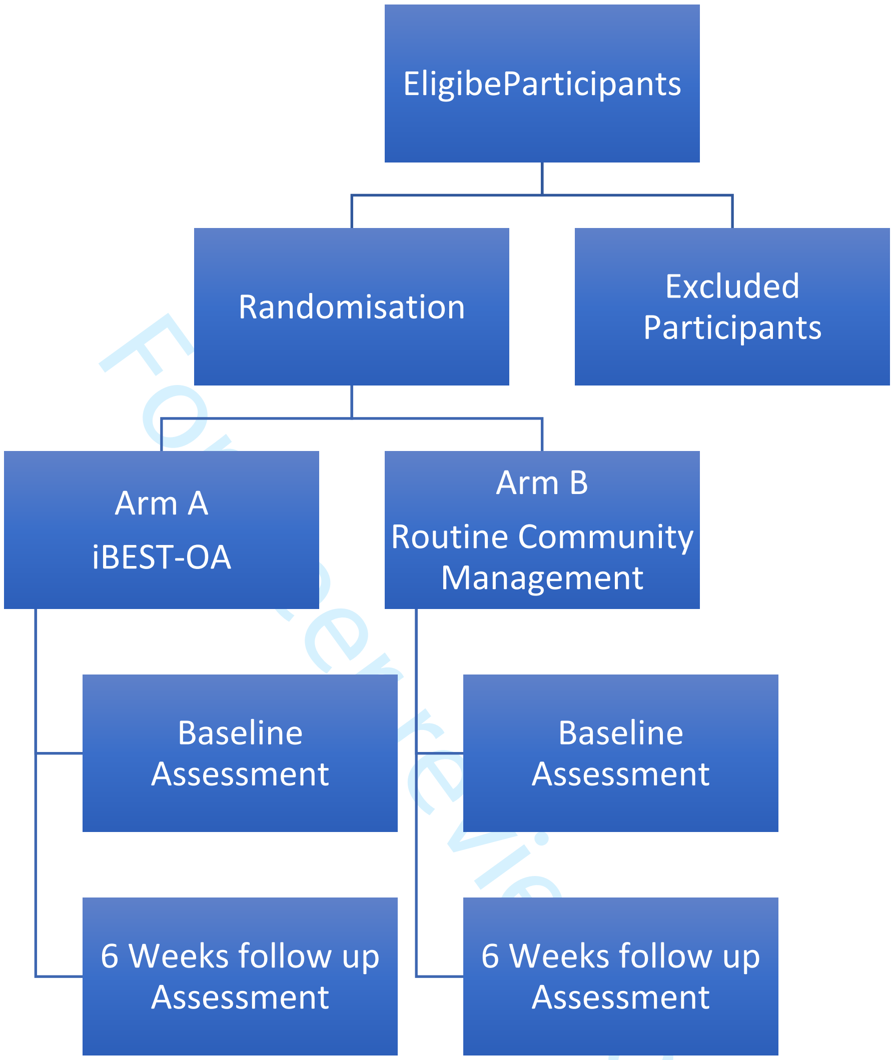

For peer review only

Figure 3: Inclusion and Exclusion Criteria for iBEAT-OA trial

INCLUSION CRITERIA

- Aged 45 years and onward
- Clinical diagnosis of knee arthritis with complaints of knee pain for 3-6 months, early morning stiffness <30 minutes, crepitus, bony tenderness, and no palpable warmth and radiographically established osteoarthritis (at least score 1 on K/L scale)
- Able to read and write English
- Able to use/access computer or tablet and have access to the internet

EXCLUSION CRITERIA

- Inability to give informed consent
- Terminal or mental illness
- Neurological conditions (Stroke, Multiple Sclerosis, Parkinson's, Motor Neuron Disease, Muscular Dystrophy, Huntington's disease), inflammatory joint diseases including rheumatoid arthritis, gout or calcium pyrophosphate deposition disease (CPPD), and dementia
- Participants with sleep apnea previously diagnosed by a physician
- Acute soft tissue injury to the knee within last 3 months before recruiting diagnosed by a physician
- Unstable heart condition or rapid fluctuations in hypertension previously diagnosed by a physician

References

1. Academy J. Better management of patients with Osteoarthritis -BOA

2008 [Available from: https://boa.registercentrum.se/boa-in-english/better-management-of-patients-with-osteoarthritis-boa/p/By_o8GxVg.

2. Peat G, McCarney R, Croft P. Knee pain and osteoarthritis in older adults: a review of community burden and current use of primary health care. Ann Rheum Dis. 2001;60(2):91-7.

3. Royal College of General P, McCormick A, Fleming D, Charlton J, Great B, Office of Population C, et al. Morbidity statistics from general practice : fourth national study 1991-1992. London: H.M.S.O.; 1995.

4. Conaghan PG, Dickson J, Grant RL. Care and management of osteoarthritis in adults: summary of NICE guidance. BMJ : British Medical Journal. 2008;336(7642):502-3.

5. Jevsevar DS. Treatment of osteoarthritis of the knee: evidence-based guideline, 2nd edition. The Journal of the American Academy of Orthopaedic Surgeons. 2013;21(9):571-6.

6. Pendleton A, Arden N, Dougados M, Doherty M, Bannwarth B, Bijlsma JW, et al. EULAR recommendations for the management of knee osteoarthritis: report of a task force of the Standing Committee for International Clinical Studies Including Therapeutic Trials (ESCISIT). Ann Rheum Dis. 2000;59(12):936-44.

7. Bennell KL, Hunter DJ, Hinman RS. Management of osteoarthritis of the knee. BMJ (Clinical research ed). 2012;345:e4934.

8. Hochberg MC, Altman RD, April KT, Benkhalti M, Guyatt G, McGowan J, et al. American College of Rheumatology 2012 recommendations for the use of nonpharmacologic and pharmacologic therapies in osteoarthritis of the hand, hip, and knee. Arthritis Care Res (Hoboken). 2012;64(4):465-74.

9. Bennell KL, Hinman RS. A review of the clinical evidence for exercise in osteoarthritis of the hip and knee. Journal of science and medicine in sport. 2011;14(1):4-9.

10. Messier SP, Loeser RF, Miller GD, Morgan TM, Rejeski WJ, Sevick MA, et al. Exercise and dietary weight loss in overweight and obese older adults with knee osteoarthritis: The arthritis, diet, and activity promotion trial. Arthritis & Rheumatism. 2004;50(5):1501-10.

11. Thomas KS, Muir KR, Doherty M, Jones AC, O'Reilly SC, Bassey EJ. Home based exercise programme for knee pain and knee osteoarthritis: randomised controlled trial. BMJ (Clinical research ed). 2002;325(7367):752.

12. van Baar ME, Assendelft WJ, Dekker J, Oostendorp RA, Bijlsma JW. Effectiveness of exercise therapy in patients with osteoarthritis of the hip or knee: a systematic review of randomized clinical trials. Arthritis and rheumatism. 1999;42(7):1361-9.

13. Chamberlain MA, Care G, Harfield B. Physiotherapy in osteoarthrosis of the knees. A controlled trial of hospital versus home exercises. International rehabilitation medicine. 1982;4(2):101-6.

14. Ettinger WH, Jr., Burns R, Messier SP, Applegate W, Rejeski WJ, Morgan T, et al. A randomized trial comparing aerobic exercise and resistance exercise with a health education program in older adults with knee osteoarthritis. The Fitness Arthritis and Seniors Trial (FAST). Jama. 1997;277(1):25-31.

15. Ettinger WH, Jr, Burns R, Messier SP, et al. A randomized trial comparing aerobic exercise and resistance exercise with a health education program in older adults with knee osteoarthritis: The fitness arthritis and seniors trial (fast). Jama. 1997;277(1):25-31.

16. Kovar PA, Allegrante JP, MacKenzie CR, Peterson MG, Gutin B, Charlson ME. Supervised fitness walking in patients with osteoarthritis of the knee. A randomized, controlled trial. Annals of internal medicine. 1992;116(7):529-34.

For peer review only

17. Minor MA, Hewett JE, Webel RR, Anderson SK, Kay DR. Efficacy of physical conditioning exercise in patients with rheumatoid arthritis and osteoarthritis. Arthritis and rheumatism. 1989;32(11):1396-405.
18. Fisher NM, Pendergast DR, Gresham GE, Calkins E. Muscle rehabilitation: its effect on muscular and functional performance of patients with knee osteoarthritis. Archives of physical medicine and rehabilitation. 1991;72(6):367-74.
19. Pisters MF, Veenhof C, van Meeteren NLU, Ostelo RW, de Bakker DH, Schellevis FG, et al. Long-Term effectiveness of exercise therapy in patients with osteoarthritis of the hip or knee: A systematic review. Arthritis Care & Research. 2007;57(7):1245-53.
20. Hurley MV, Walsh NE, Mitchell HL, Pimm TJ, Patel A, Williamson E, et al. Clinical effectiveness of a rehabilitation program integrating exercise, self-management, and active coping strategies for chronic knee pain: A cluster randomized trial. Arthritis Care & Research. 2007;57(7):1211-9.
21. Dunlop DD, Song J, Semanik PA, Sharma L, Chang RW. Physical activity levels and functional performance in the osteoarthritis initiative: a graded relationship. Arthritis and rheumatism. 2011;63(1):127-36.
22. Esser S, Bailey A. Effects of exercise and physical activity on knee osteoarthritis. Current pain and headache reports. 2011;15(6):423-30.
23. Lange AK, Vanwanseele B, Fiatarone Singh MA. Strength training for treatment of osteoarthritis of the knee: a systematic review. Arthritis and rheumatism. 2008;59(10):1488-94.
24. Mikesky AE, Mazzuca SA, Brandt KD, Perkins SM, Damush T, Lane KA. Effects of strength training on the incidence and progression of knee osteoarthritis. Arthritis Care & Research. 2006;55(5):690-9.
25. Baker K, McAlindon T. Exercise for knee osteoarthritis. Current opinion in rheumatology. 2000;12(5):456-63.
26. Peeler J, Ripat J. The effect of low-load exercise on joint pain, function, and activities of daily living in patients with knee osteoarthritis. The Knee. 2018.
27. UK AR. Exercises to manage Knee Pain [Available from: https://www.arthritisresearchuk.org/arthritis-information/exercises-to-manage-pain/knee-pain-exercises.aspx.
28. BOA. Better management of patients with OsteoArthritis (BOA) [Available from: https://boa.registercentrum.se/boa-in-english/better-management-of-patients-with-osteoarthritis-boa/p/By_o8GxVg.
29. Thorstensson CA, Garellick G, Rystedt H, Dahlberg LE. Better Management of Patients with Osteoarthritis: Development and Nationwide Implementation of an Evidence-Based Supported Osteoarthritis Self-Management Programme. Musculoskeletal care. 2015;13(2):67-75.
30. Talbot LA, Gaines JM, Huynh TN, Metter EJ. A Home-Based Pedometer-Driven Walking Program to Increase Physical Activity in Older Adults with Osteoarthritis of the Knee: A Preliminary Study. Journal of the American Geriatrics Society. 2003;51(3):387-92.
31. Lorig KR, Ritter PL, Laurent DD, Plant K. The internet-based arthritis self-management program: A one-year randomized trial for patients with arthritis or fibromyalgia. Arthritis Care & Research. 2008;59(7):1009-17.
32. Bossen D, Veenhof C, Van Beek KEC, Spreeuwenberg PMM, Dekker J, De Bakker DH. Effectiveness of a Web-Based Physical Activity Intervention in Patients With Knee and/or Hip Osteoarthritis: Randomized Controlled Trial. Journal of Medical Internet Research. 2013;15(11):e257.
33. Brooks MA, Beaulieu JE, Severson HH, Wille CM, Cooper D, Gau JM, et al. Web-based therapeutic exercise resource center as a treatment for knee osteoarthritis: a prospective cohort pilot study. BMC musculoskeletal disorders. 2014;15:158.
34. Dahlberg LE, Grahn D, Dahlberg JE, Thorstensson CA. A Web-Based Platform for Patients With Osteoarthritis of the Hip and Knee: A Pilot Study. JMIR research protocols. 2016;5(2):e115.

For peer review only

35. Nero H, Dahlberg J, Dahlberg EL. A 6-Week Web-Based Osteoarthritis Treatment Program: Observational Quasi-Experimental Study. J Med Internet Res. 2017;19(12):e422.
36. Wilcox S, Brenes GA, Levine D, Sevick MA, Shumaker SA, Craven T. Factors related to sleep disturbance in older adults experiencing knee pain or knee pain with radiographic evidence of knee osteoarthritis. J Am Geriatr Soc. 2000;48(10):1241-51.
37. Power JD, Perruccio AV, Badley EM. Pain as a mediator of sleep problems in arthritis and other chronic conditions. Arthritis Care & Research. 2005;53(6):911-9.
38. Smith MT, Quartana PJ, Okonkwo RM, Nasir A. Mechanisms by which sleep disturbance contributes to osteoarthritis pain: a conceptual model. Current pain and headache reports. 2009;13(6):447-54.
39. Leigh TJ, Hindmarch I, Bird HA, Wright V. Comparison of sleep in osteoarthritic patients and age and sex matched healthy controls. Ann Rheum Dis. 1988;47(1):40-2.
40. Doherty M, Smith J. Elusive 'alpha-delta' sleep in fibromyalgia and osteoarthritis. Ann Rheum Dis. 1993;52(3):245.
41. Harrison MM, Childs A, Carson PE. Incidence of undiagnosed sleep apnea in patients scheduled for elective total joint arthroplasty. The Journal of arthroplasty. 2003;18(8):1044-7.
42. Association ASD. International classification of sleep disorders. Revised: Diagnostic and coding manual. http://www absm org/PDF/ICSD pdf. 1997.
43. Campbell CM, Buenaver LF, Finan P, Bounds SC, Redding M, McCauley L, et al. Sleep, Pain Catastrophizing, and Central Sensitization in Knee Osteoarthritis Patients With and Without Insomnia. Arthritis Care Res (Hoboken). 2015;67(10):1387-96.
44. Gosset M, Berenbaum F, Salvat C, Sautet A, Pigenet A, Tahiri K, et al. Crucial role of visfatin/pre-B cell colony-enhancing factor in matrix degradation and prostaglandin E2 synthesis in chondrocytes: possible influence on osteoarthritis. Arthritis and rheumatism. 2008;58(5):1399-409.
45. Sowers M, Karvonen-Gutierrez CA, Palmieri-Smith R, Jacobson JA, Jiang Y, Ashton-Miller JA. Knee osteoarthritis in obese women with cardiometabolic clustering. Arthritis Care and Research. 2009;61(10):1328-36.
46. Puenpatom RA, Victor TW. Increased prevalence of metabolic syndrome in individuals with osteoarthritis: an analysis of NHANES III data. Postgraduate medicine. 2009;121(6):9-20.
47. Wang X, Hunter D, Xu J, Ding C. Metabolic triggered inflammation in osteoarthritis. Osteoarthritis and Cartilage. 2015;23(1):22-30.
48. Zhuo Q, Yang W, Chen J, Wang Y. Metabolic syndrome meets osteoarthritis. Nature Reviews Rheumatology. 2012;8:729.
49. Gregor MF, Hotamisligil GS. Inflammatory mechanisms in obesity. Annual review of immunology. 2011;29:415-45.
50. Fernandes GS, Sarmanova A, Warner S, Harvey H, Akin-Akinyosoye K, Richardson H, et al. Knee pain and related health in the community study (KPIC): a cohort study protocol. BMC musculoskeletal disorders. 2017;18(1):404-.
51. Fransen M, McConnell S, Harmer AR, Van der Esch M, Simic M, Bennell KL. Exercise for osteoarthritis of the knee: a Cochrane systematic review. British Journal of Sports Medicine. 2015;49(24):1554-7.
52. GJ ETD. Closed Kinetic Chain Exercise: A Comprehensive Guide to Multiple Joint Exercise: Human Kinetics Publishers; 2001. 128 pages p.
53. Ferreira-Valente MA, Pais-Ribeiro JL, Jensen MP. Validity of four pain intensity rating scales. PAIN®. 2011;152(10):2399-404.
54. Hjermstad MJ, Fayers PM, Haugen DF, Caraceni A, Hanks GW, Loge JH, et al. Studies Comparing Numerical Rating Scales, Verbal Rating Scales, and Visual Analogue Scales for Assessment of Pain Intensity in Adults: A Systematic Literature Review. Journal of Pain and Symptom Management. 2011;41(6):1073-93.
55. Karcioglu O, Topacoglu H, Dikme O, Dikme O. A systematic review of the pain scales in adults: Which to use? The American Journal of Emergency Medicine. 2018.

For peer review only

56. McConnell S, Kolopack P, Davis AM. The Western Ontario and McMaster Universities Osteoarthritis Index (WOMAC): a review of its utility and measurement properties. Arthritis Care & Research. 2001;45(5):453-61.
57. Bellamy N, Buchanan WW, Goldsmith CH, Campbell J, Stitt LW. Validation study of WOMAC: a health status instrument for measuring clinically important patient relevant outcomes to antirheumatic drug therapy in patients with osteoarthritis of the hip or knee. J Rheumatol. 1988;15(12):1833-40.
58. Hill JC, Kang S, Benedetto E, Myers H, Blackburn S, Smith S, et al. Development and initial cohort validation of the Arthritis Research UK Musculoskeletal Health Questionnaire (MSK-HQ) for use across musculoskeletal care pathways. BMJ Open. 2016;6(8).
59. Hill J, Kang S, Benedetto E, Myers H, Blackburn S, Smith S, et al. PMS74 - Development of the Musculoskeletal Health Questionnaire (MSK-HQ) for Use in Different Conditions and Different Healthcare Pathways. Value in Health. 2016;19(7):A544.
60. Backhaus J, Junghanns K, Broocks A, Riemann D, Hohagen F. Test–retest reliability and validity of the Pittsburgh Sleep Quality Index in primary insomnia. Journal of Psychosomatic Research. 2002;53(3):737-40.
61. Buysse DJ, Reynolds IIICF, Monk TH, Hoch CC, Yeager AL, Kupfer DJ. Quantification of Subjective Sleep Quality in Healthy Elderly Men and Women Using the Pittsburgh Sleep Quality Index (PSQI). Sleep. 1991;14(4):331-8.
62. Carpenter JS, Andrykowski MA. Psychometric evaluation of the pittsburgh sleep quality index. Journal of Psychosomatic Research. 1998;45(1):5-13.
63. Finan PH, Buenaver LF, Bounds SC, Hussain S, Park RJ, Haque UJ, et al. Discordance between pain and radiographic severity in knee osteoarthritis: Findings from quantitative sensory testing of central sensitization. Arthritis & Rheumatism. 2013;65(2):363-72.
64. Gill S, McBurney H. Reliability of performance-based measures in people awaiting joint replacement surgery of the hip or knee. Physiotherapy research international : the journal for researchers and clinicians in physical therapy. 2008;13(3):141-52.
65. Gill SD, de Morton NA, Mc Burney H. An investigation of the validity of six measures of physical function in people awaiting joint replacement surgery of the hip or knee. Clinical rehabilitation. 2012;26(10):945-51.
66. Jones CJ, Rikli RE, Beam WC. A 30-s chair-stand test as a measure of lower body strength in community-residing older adults. Research quarterly for exercise and sport. 1999;70(2):113-9.
67. Bennell K, Dobson F, Hinman R. Measures of physical performance assessments: Self-Paced Walk Test (SPWT), Stair Climb Test (SCT), Six-Minute Walk Test (6MWT), Chair Stand Test (CST), Timed Up & Go (TUG), Sock Test, Lift and Carry Test (LCT), and Car Task. Arthritis Care & Research. 2011;63(S11):S350-S70.
68. Podsiadlo D, Richardson S. The Timed "Up & Go": A Test of Basic Functional Mobility for Frail Elderly Persons. Journal of the American Geriatrics Society. 1991;39(2):142-8.
69. Arendt-Nielsen L, Nie H, Laursen MB, Laursen BS, Madeleine P, Simonsen OH, et al. Sensitization in patients with painful knee osteoarthritis. Pain. 2010;149(3):573-81.
70. Finan PH, Buenaver LF, Bounds SC, Hussain S, Park RJ, Haque UJ, et al. Discordance between pain and radiographic severity in knee osteoarthritis: findings from quantitative sensory testing of central sensitization. Arthritis and rheumatism. 2013;65(2):363-72.
71. Imamura M, Imamura ST, Kaziyama HH, Targino RA, Hsing WT, de Souza LP, et al. Impact of nervous system hyperalgesia on pain, disability, and quality of life in patients with knee osteoarthritis: a controlled analysis. Arthritis and rheumatism. 2008;59(10):1424-31.
72. Lee YC, Lu B, Bathon JM, Haythornthwaite JA, Smith MT, Page GG, et al. Pain sensitivity and pain reactivity in osteoarthritis. Arthritis Care Res (Hoboken). 2011;63(3):320-7.
73. Neogi T, Frey-Law L, Scholz J, Niu J, Arendt-Nielsen L, Woolf C, et al. Sensitivity and sensitisation in relation to pain severity in knee osteoarthritis: trait or state? Ann Rheum Dis. 2015;74(4):682-8.

For peer review only

74. Neogi T, Guermazi A, Roemer F, Nevitt MC, Scholz J, Arendt-Nielsen L, et al. Association of Joint Inflammation With Pain Sensitization in Knee Osteoarthritis: The Multicenter Osteoarthritis Study. Arthritis & rheumatology (Hoboken, NJ). 2016;68(3):654-61.
75. Wessel J. The reliability and validity of pain threshold measurements in osteoarthritis of the knee. Scandinavian journal of rheumatology. 1995;24(4):238-42.
76. D'Agostino MA, Conaghan P, Le Bars M, Baron G, Grassi W, Martin-Mola E, et al. EULAR report on the use of ultrasonography in painful knee osteoarthritis. Part 1: Prevalence of inflammation in osteoarthritis. Annals of the Rheumatic Diseases. 2005;64(12):1703-9.
77. Hayashi D, Roemer FW, Katur A, Felson DT, Yang S-O, Alomran F, et al. Imaging of Synovitis in Osteoarthritis: Current Status and Outlook. Seminars in Arthritis and Rheumatism. 2011;41(2):116-30.
78. Karim Z, Wakefield RJ, Quinn M, Conaghan PG, Brown AK, Veale DJ, et al. Validation and reproducibility of ultrasonography in the detection of synovitis in the knee: a comparison with arthroscopy and clinical examination. Arthritis and rheumatism. 2004;50(2):387-94.
79. Sarmanova A, Hall M, Fernandes GS, Bhattacharya A, Valdes AM, Walsh DA, et al. Association between ultrasound-detected synovitis and knee pain: a population-based case-control study with both cross-sectional and follow-up data. Arthritis research & therapy. 2017;19(1):281.
80. Sarmanova A, Hall M, Moses J, Doherty M, Zhang W. Synovial changes detected by ultrasound in people with knee osteoarthritis – a meta-analysis of observational studies. Osteoarthritis and Cartilage. 2016;24(8):1376-83.
81. Franchi MV, Longo S, Mallinson J, Quinlan JI, Taylor T, Greenhaff PL, et al. Muscle thickness correlates to muscle cross-sectional area in the assessment of strength training-induced hypertrophy. Scandinavian journal of medicine & science in sports. 2017.
82. Strasser EM, Draskovits T, Praschak M, Quittan M, Graf A. Association between ultrasound measurements of muscle thickness, pennation angle, echogenicity and skeletal muscle strength in the elderly. AGE. 2013;35(6):2377-88.
83. Tan J, Balci N, Sepici V, Gener FA. Isokinetic and isometric strength in osteoarthrosis of the knee. A comparative study with healthy women. Am J Phys Med Rehabil. 1995;74(5):364-9.
84. Berry PA, Jones SW, Cicuttini FM, Wluka AE, Maciewicz RA. Temporal relationship between serum adipokines, biomarkers of bone and cartilage turnover, and cartilage volume loss in a population with clinical knee osteoarthritis. Arthritis & Rheumatism. 2011;63(3):700-7.
85. Richette P, Poitou C, Garnero P, Vicaut E, Bouillot J-L, Lacorte J-M, et al. Benefits of massive weight loss on symptoms, systemic inflammation and cartilage turnover in obese patients with knee osteoarthritis. Annals of the Rheumatic Diseases. 2011;70(1):139-44.
86. Excellence NIfHaC. Osteoarthritis: care and management 2014 [Available from: https://www.nice.org.uk/guidance/cg177/chapter/1-Recommendations#non-pharmacological-management-2.
87. Campbell R, Evans M, Tucker M, Quilty B, Dieppe P, Donovan JL. Why don't patients do their exercises? Understanding non-compliance with physiotherapy in patients with osteoarthritis of the knee. Journal of Epidemiology and Community Health. 2001;55(2):132-8.
88. Jack K, McLean SM, Moffett JK, Gardiner E. Barriers to treatment adherence in physiotherapy outpatient clinics: A systematic review. Manual Therapy. 2010;15(3):220-8.
89. Marks R, Allegrante JP. Chronic osteoarthritis and adherence to exercise: a review of the literature. Journal of aging and physical activity. 2005;13(4):434-60.
90. Eytan T, Benabio J, Golla V, Parikh R, Stein S. Social Media and the Health System. The Permanente Journal. 2011;15(1):71-4.
91. Moorhead SA, Hazlett DE, Harrison L, Carroll JK, Irwin A, Hoving C. A New Dimension of Health Care: Systematic Review of the Uses, Benefits, and Limitations of Social Media for Health Communication. Journal of Medical Internet Research. 2013;15(4):e85.
92. Thackeray R, Crookston BT, West JH. Correlates of Health-Related Social Media Use Among Adults. Journal of Medical Internet Research. 2013;15(1):e21.

For peer review only

Authors' contributions:

Sameer Gohir is the primary author and all other authors are secondary. Dr Ana M. Valdes is the main supervisor and leading this project.

Funding statement:

This study is part-funded by the Arthritis Research UK Pain Centre (University of Nottingham) and part-funded by Nottingham Biomedical Research Centre (BRC).

Competing interests statement:

None Declared

For peer review only

Figure 1: A randomised controlled trial evaluating the efficacy of internet-Based Exercises Aimed at Treating Knee Osteoarthritis (iBEAT-OA) – PICOT Format

Research Question

- Will iBEAT-OA reduce the knee pain?

Population

- Age 45 and above, male or female and suffering from knee osteoarthritis

Intervention

- Internet bases exercises aimed at treating knee OA (iBEAT-OA)

Comparison

- Participants with knee OA and following routine management for knee OA in the community

Outcome Measures

- NRS
- Sleep
- QST
- MSK-USS
- MTA of Vastus Lateralis Oblique (VLO) using MSK-USS
- MVC of Quadriceps
- TUG
- 30CST
- WOMAC
- PSQI
- MSK- HQ
- BIA

Time

- 6 weeks

*NRS= Numerical Rating Scale, QST= Quantitative Sensory Testing, MSK USS= Musculoskeletal Ultrasound, MTA =Muscle Thickness Assessment, MVC= Maximum Voluntary Contraction- isokinetic contraction of Quadriceps muscle, TUG = Time up and go test, 30CST= 30-second sit to stand test, WOMAC= Western Ontario and McMaster Universities Osteoarthritis Index, PSQI= Pittsburgh Sleep Quality Index, MSK-HQ= Arthritis Research UK Musculoskeletal Health Questionnaire, BIA = Bioimpedance Assessment

For peer review only

Figure 2: iBEAT-OA Trial design. The flowchart summarises the design of iBEAT-OA trial.

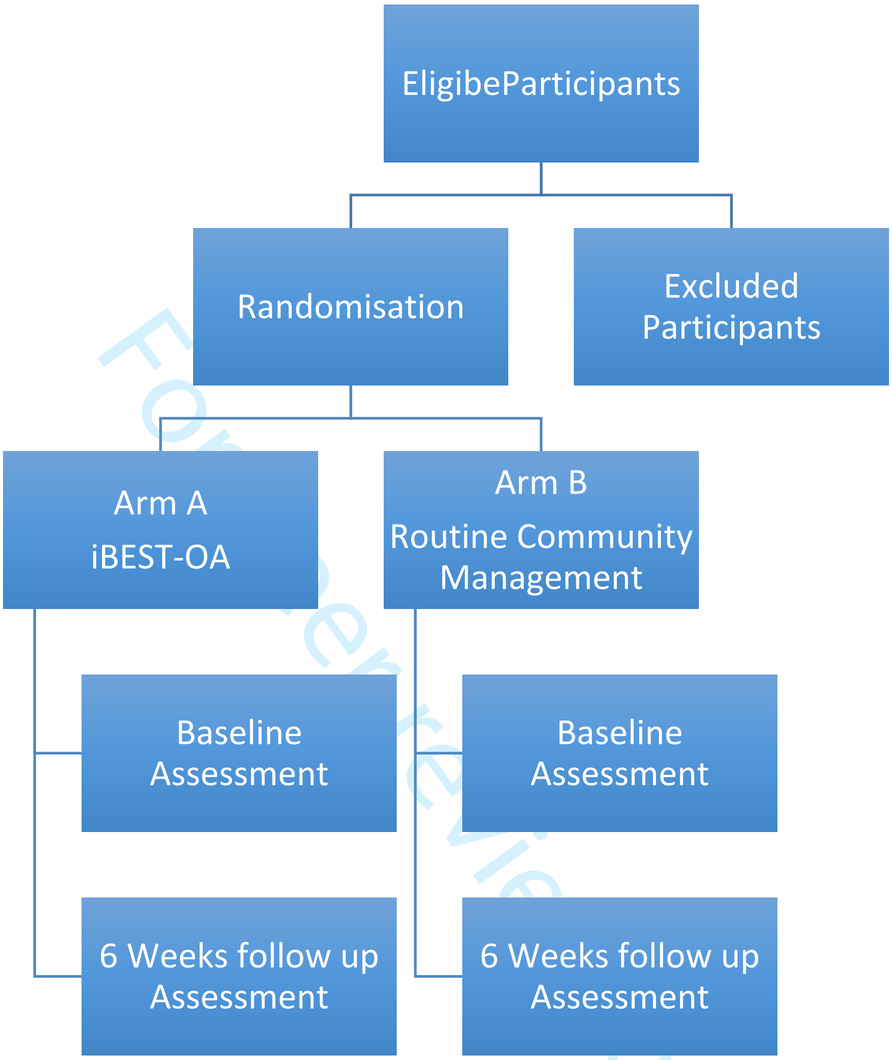

For peer review only

Figure 3: Inclusion and Exclusion Criteria for iBEAT-OA trial

INCLUSION CRITERIA

- Aged 45 years and onward
- Clinical diagnosis of knee arthritis with complaints of knee pain for 3-6 months, early morning stiffness <30 minutes, crepitus, bony tenderness, and no palpable warmth and radiographically established osteoarthritis (at least score 1 on K/L scale)
- Able to read and write English
- Able to use/access computer or tablet and have access to the internet

EXCLUSION CRITERIA

- Inability to give informed consent
- Terminal or mental illness
- Neurological conditions (Stroke, Multiple Sclerosis, Parkinson’s, Motor Neuron Disease, Muscular Dystrophy, Huntington’s disease), inflammatory joint diseases including rheumatoid arthritis, gout or calcium pyrophosphate deposition disease (CPPD), and dementia
- Participants with sleep apnea previously diagnosed by a physician
- Acute soft tissue injury to the knee within last 3 months before recruiting diagnosed by a physician
- Unstable heart condition or rapid fluctuations in hypertension previously diagnosed by a physician

A study protocol for a randomised controlled trial evaluating the efficacy of an Internet-Based Exercise programme Aimed at Treating Knee Osteoarthritis (iBEAT-OA) in the community

Journal:	*BMJ Open*
Manuscript ID	bmjopen-2019-030564.R1
Article Type:	Protocol
Date Submitted by the Author:	22-Jul-2019
Complete List of Authors:	Gohir, Sameer; University of Nottingham Division of Academic Rheumatology,<br>Greenhaff, Paul; University of Nottingham School of Medical and Surgical Sciences, Division of Physiology, Pharmacology and Neuroscience<br>Abhishek, A; University of Nottingham Faculty of Medicine and Health Sciences, Rheumatology, Faculty of Medicine & Health Sciences<br>Valdes, A; University of Nottingham Faculty of Medicine and Health Sciences
**Primary Subject Heading**:	Rehabilitation medicine
Secondary Subject Heading:	Sports and exercise medicine
Keywords:	knee osteoarthritis, web based intervention, iBEAT-OA, knee pain

A study protocol for a randomised controlled trial evaluating the efficacy of an Internet-Based Exercise programme Aimed at Treating Knee Osteoarthritis (iBEAT-OA) in the community

Sameer Gohir[1*], Paul Greenhaff[1,2,3], Abhishek Abhishek[1,4], Ana Valdes[1]

[1]NIHR Nottingham Biomedical Research Centre, School of Medicine, University of Nottingham, [2]MRC Arthritis Research UK Centre for MSK Ageing Research, [3]School of Life Sciences, University of Nottingham, [4]Academic Rheumatology, University of Nottingham

(Corresponding Author: Sameer Gohir and e-mail: sameer.gohir@nottingham.ac.uk)*

Abstract

Introduction

Knee osteoarthritis (OA) is the most common joint disease worldwide. As of today, there are no disease-modifying drugs, but there is evidence that muscle strengthening exercises can substantially reduce pain and improve function in this disorder, and one very well tested physiotherapy protocol is the "Better Management of Patients with Osteoarthritis" (BOA) developed in Sweden. Given the high prevalence of knee OA, a potentially cost-effective digital delivered approach to treat knee OA should be trialled. This study aims to explore the benefits of an Internet-Based Exercise Programme Aimed at Treating knee OA (iBEAT-OA) in modulating pain, function and other health-related outcomes in individuals with knee OA.

Methods and Analysis

A randomised controlled trial (RCT) was designed to evaluate the efficacy of a web-based exercise programme in a population with knee OA compared with standard community care provided by general practitioners (GP) in the UK. We anticipate recruiting participants into equal groups. The intervention group (n=67) will exercise for 20-30 minutes daily for six consecutive weeks whereas the control group (n=67) will follow GP recommended routine care. The participants will be assessed using a Numerical Rating Scale (NRS), the Western Ontario and McMaster Universities Osteoarthritis Index (WOMAC), the Arthritis Research UK Musculoskeletal Health Questionnaire (MSK-HQ), the Pittsburgh Sleep Quality Index (PSQI), 30-second sit to stand test (30CST), time to up and go test (TUG), Quantitative Sensory Testing (QST), musculoskeletal ultrasound scan (MSK-USS), Muscle Thickness Assessment (MTA) of the vastus lateralis, quadriceps muscles force generation during an isokinetic Maximum Voluntary Contraction (MVC). Samples of urine, blood, faeces and synovial fluid will be collected to establish biomarkers associated with changes in pain and sleep patterns in individuals affected with knee OA. Standard parametric regression methods will be used for statistical analysis.

Ethics and Dissemination

Ethical approval was obtained from the Research Ethics Committee (REC) (Ref: 18/EM/0154) and the Health Research Authority (HRA) (Protocol no: 18021). The Trial Registration Number for this study is

For peer review only - http://bmjopen.bmj.com/site/about/guidelines.xhtml

NCT03545048 (Registered in June 2018). The results of the trial will be submitted for publication in a peer-reviewed journal.

Strength and Limitation of this Study

- This study is the first to evaluate a web-based exercise intervention to improve pain in sufferers of knee OA in the United Kingdom.
- We plan to recruit only those individuals presenting with radiographic evidence of knee OA that will rule out non-OA causes of knee pain.
- This study is the first study to evaluate changes in sleep disturbance due to knee OA along with an exercise intervention.
- The lack of double-blind study design is typically a limitation of exercise and lifestyle intervention studies.
- Six weeks iBEAT-OA intervention may not show a statistically detectable reduction in knee pain and may be a potential limitation of this study.

Introduction

Knee OA is a common cause of disability globally, and is mostly managed in primary care (1). In the United Kingdom, 10% people between the ages of 65 and 74 consult their general practitioners (GPs) for OA every year (2). In the UK, one in twenty-five, people consult their GP for knee OA annually (1).

The first line of treatment for OA involves exercise, education and weight loss if applicable(3-7). Exercise improves joint and patient centred outcomes in knee OA, and encourages them to manage activities of daily living (8-22). In fact, strengthening knee exercises have been shown to reduce progression of radiographic changes of knee OA (23). A recent Cochrane review on exercise interventions for Knee OA has reported that exercise significantly reduced pain (12 points/100; 95% CI 10-15) and improved physical function (10 points/100; 95% CI 8-13) to a moderate degree (24). Another systemic review and meta-analysis conducted on the effect of resistance exercises in patients with knee osteoarthritis reported that resistance training relieved pain (Standard Mean Difference [SMD]: -0.43; 95% CI: -0.57 to -0.29) (25). There is a disparity of opinion regarding the effectiveness of different types of exercise for knee OA to reduce the pain, and a combination of open and closed isotonic exercises are recommended for knee OA (26). Symptoms of knee arthritis can be exacerbated by following ineffective or unsafe exercises regimens (27-30), leading to poor prognosis and poor adherence to exercise intervention (31). Hence the need to choose the type and frequency of exercise intervention carefully.

Versus Arthritis recommend knee exercise for 'knee pain', however these exercises are not specific to knee OA and include soft tissue injuries of knee as a cause for knee pain (32). An exercise regimen is normally recommended by GPs as a part of the first line of treatment when they consult someone with arthritis-related knee pain. If an exercise intervention fails to impact positively on pain perception, patients are referred for Physiotherapy. After a comprehensive assessment by physiotherapists, a variety of exercise interventions can be prescribed that fluctuate in exercise modality, intensity, and duration, but with variable success in terms of outcome. Therefore, there is a need for standardised exercise interventions to address knee OA pain, which would ideally be accessible online to maximise efficacy and cost-effectiveness. iBEAT-OA is the first randomised controlled trial conducted on the App-based knee exercises developed by Joint Academy (JA). The exercise intervention (JA App), which we will use in this project, is a web-based programme derived from the Supported Osteoarthritis Self-Management Programme (SOASP) also known as "Better Management of Patients with Osteoarthritis" (BOA) developed in Sweden(33). Essentially, the JA

For peer review only - http://bmjopen.bmj.com/site/about/guidelines.xhtml

App is a digital version of face-to-face BOA. From January 2008 until January 2017, around 75,000 patients participated in the SOASP, 2339 physiotherapists and occupational therapists were educated to deliver the Supported Osteoarthritis Self-Management Programme, and today SOASP is offered in 700 clinics all over Sweden (33). This intervention has been rated good to very good by 94% of patients at reducing pain related to knee OA and has an excellent safety profile and acceptability (34). A Quasi study done on internet based Joint Academy programme has shown a change in mean numerical rating scale (NRS) which was larger than the minimal clinical difference (5.4 vs 4.1; P<.001) (35).

There exist a number of other exercise programmes within care programmes for people with knee OA pain. Among these are the:

Good Life with OA in Denmark (GLA:D) programme, which is a registry based study that implemented clinical guidelines (patient education and exercise but not weight loss) to knee OA patients (36). The programme consists of a 2-day training course for physiotherapists (PTs), including training to diagnose and deliver OA care and an 8-week supervised exercise intervention for OA patients, with a minimum intervention of 3 sessions in total. However, GLA:D does not deliver intervention via a web or smartphone App.

The ESCAPE (Enabling Self-management and Coping with Arthritic Pain using Exercise) app is a support tool for people who have already attended a person-to-person ESCAPE programme (19) and enables participants to continue to exercise safely in the home environment following the person-to-person programme. A limitation of this approach is that the app is a support tool for the main programme and provided for reminding exercise. It cannot be used as the sole basis for treatment and care of knee OA. Their recommendations are to use it in conjunction with the advice and professional judgment of GP or other healthcare practitioner, which is the limitation of this App.

Because of the high prevalence of knee OA, strategies to deliver exercise interventions that are both efficacious and inexpensive should be prioritised. Some of the issues relating to the delivery of exercise interventions for knee OA include compliance, accessibility to clinics for people with mobility problems and the cost of delivering such services. There are previous studies which have assessed the efficacy of home-based exercises (10, 12, 37), however, very few studies looked at web-based delivery of exercise intervention (35, 38-43). Most of these studies recruited patients with knee pain, and only two studies assessed for radiographic evidence of knee OA (40, 42). This makes the results of these studies less specific to base a recommended treatment for knee OA. In addition, none of these studies has investigated the role of exercise interventions for pain relief, assessing levels of pain sensitivity and central sensitisation, which are likely different for knee OA and other causes (e.g. soft tissue injuries) of knee pain.

The most recent RCT which studied the benefit of internet based exercises versus routine physiotherapy reported no difference on WOMAC at 12 months between internet-based vs face to face physiotherapy and suggested further studies with strategies to maximise the benefit of exercises based interventions for patients with knee OA (42). Therefore, there is a need for specific, efficacious, and cost effective exercise programme that individuals can perform in the home settings.

Knee OA pain is accompanied by a number of additional disturbances that influence the individual's health, which we also propose to study. There is a well-known link between sleep disturbance and chronic pain, and epidemiological studies (44-46) and experimental studies (47-51) have established a link between disturbed sleep and knee OA. These studies suggest that individuals with OA have

For peer review only - http://bmjopen.bmj.com/site/about/guidelines.xhtml

greater sleep disturbances. Furthermore, sleep disturbance is recognised as an important factor in determining pain perception (51). A relationship between sleep disturbance and pain severity in knee OA patients is usually explored, and sleep disturbances such as shortened sleep duration and fragmented sleep (44) have been associated with increased sensitivity to pain in OA patients, and consequently with decreased quality of life (46). Therefore, studying the sleeping pattern is highly relevant if we are discussing a successful online exercise programme for knee OA.

Recent epidemiological and clinical studies have also underlined that metabolic syndrome (MetS) has the most significant impact on the initiation and severity of OA (52-56). Metabolic triggered inflammation, also known as meta-inflammation (57) can be a result of abnormalities in body composition, adipokines, cytokines, lipids, and vitamin D and has been associated with the pathogenesis of OA (55). Given that body composition is influenced by a number of the variables associated with OA, e.g., systemic inflammation, sleep patterns, altered physical activity levels, and altered energy levels, it is important to study this as well. The intricate links between exercises, sleep, pain, metabolic syndrome, body composition, and OA are not fully understood. To date, the relationship between improvements in pain due to exercise for pain relief and other changes related to health parameters that have been linked to OA or chronic pain has not been explored. Specifically, the effects of exercise for knee pain relief on sleep, biomarkers of inflammation and insulin resistance is unknown. We aim to study these parameters and establish the link between exercises for knee OA and these parameters. Figure 1 shows this in PICOT format.

The efficacy of web-based delivery of exercises has not been evaluated in a UK setting. This is first study to conduct web-based exercises intervention in the United Kingdom. This study is also novel because it will be the first in the UK to bring together a wide range of factors influencing Knee OA. This will include QST, which is deemed to be an objective measure along with measurement of sleep patterns undertaken using an activity monitor (Actigraphy).

Rationale

This study aims to explore the benefits of an internet-based exercise programme in patients with knee OA to establish if six week intervention reduces pain perception and sensitivity. Being a web-based exercise intervention, also makes it accessible and cost-effective to volunteers and physiotherapists, and will hopefully help establish a standardised intervention for knee OA that will maintain individual motivation, compliance, and managing pain. The study will also endeavour to explore the complicated relationship between chronic pain, sleep, biomarkers of inflammation, body composition, and knee OA. Therefore, this study could potentially help generate recommendations for the treatment of knee OA.

Primary Objective

To test whether an internet-based exercise intervention can reduce pain perception (NRS) in knee OA.

Secondary Objective

To test whether there is a benefit of iBEAT-OA to improve sleep disturbances, reduce pain sensitisation and metabolic syndrome.

For peer review only - http://bmjopen.bmj.com/site/about/guidelines.xhtml

Methods and Analysis

iBEAT-OA Trial Design

The iBEAT-OA study is a randomised controlled trial in the primary care setting in Nottingham with participants identified as having knee OA, 1:1 randomised to web-based exercises or usual care as shown in Figure 2.

Setting

Home based - Primary Care Setting

Patient and Public Involvement

At the patient and public involvement (PPI) representative meeting of 14/12/2015, seven volunteers suffering from chronic OA pain were asked about the relevance of studying sleep in the context of OA pain. They all were very supportive of studying and understanding sleep patterns. They viewed actigraphy as a good non-invasive alternative to polysomnography and saw no problem with using the device for six weeks.

On 17/05/2017 six representatives from the PPI Musculoskeletal group were asked about exercise interventions and were all supportive of this. They were also asked about the extraction of synovial fluid from their joints. Five out of six said they would not have a problem with this if it was performed by a specialist using ultrasound to guide the needle, this approach was therefore adopted as an optional test within the study.

The latest version of the patient facing documentation (Participant information sheet, consent form, invitation letter and recruitment flyer) have been shared with the PPI group and received favourable comments.

Recruitment

A selection of eligible people for the study will be invited from existing databases (58) held at Academic Rheumatology, City Hospital Nottingham of participants with knee pain who have agreed to be contacted for future studies. The inclusion and exclusion criteria are shown in Figure 3. Any shortfall in the recruitment will be compensated by sending study leaflets to GP surgeries. The GPs will follow the inclusion and exclusion criteria. All those individuals who are suitable for the study will be sent the study information sheet. Those who return the completed screening consent form will be contacted and screened for inclusion in the study.

Randomisation and blinding

The eligible participants will undergo computer-generated randomisation and allocation concealment. Randomisation will be done using “sealed envelope” (https://www.sealedenvelope.com/), an online randomisation service. Participants will be equally allocated between treatment arms with at least n=67 per arm and this will be done by the research team. At this point, individuals will be alerted to which study group they have been assigned and hence blinding is not possible. Because this might affect the motivation of participants randomised into the control arm, these individuals will be offered access to the web-based exercise programme after completing the study. Blinding of exercise intervention studies is difficult; however the clinical research team will be blinded to the intervention

For peer review only - http://bmjopen.bmj.com/site/about/guidelines.xhtml

Sample size and justification

A Swedish study using specifically Joint Academy as the intervention and NRS as the outcome reported an effect size of 1.3 on the NRS (35), which corresponds to 0.56 SD (average SD 2.3). A study with an expected 60 participants per arm at the end of the 6-week intervention has 86% power to achieve such an effect size with an alpha of 0.05. However, a recent systematic review of 44 high-quality exercise trials for knee OA pain (3537 participants) (24) found an average effect of 12/100 VAS points corresponding to 0.49 standard deviations. A sample size of n=60 per group is necessary to achieve 75% statistical power. The estimated dropout rate for exercise interventions is 10%, therefore, a sample of 67 per arm will be recruited to achieve between 75%-86% power, i.e. 80% power with an alpha level of 0.05.

Intervention

iBEAT-OA will use a web-based exercise platform known as Joint Academy (JA) as recent pilot studies (35, 41) demonstrated promising results. This programme is based on a Swedish face to face self-management program known as ‘Artrosskolan’ (*The Osteoarthritis School*), which provided structured information and exercises for knee OA to a relevant population suffering from knee OA. This was initially introduced as “Better Management of Patients with Osteoarthritis” (BOA) (59). The compliance of this self-management programme has been reported as good. 62% of 20,200 patients reported daily use of this programme at three months follow-up however, this percentage drops to 37% at 12 months review (34). The company that produced the Joint Academy platform has given permission to use their web-based App to conduct this study.

The exercise intervention comprises a mixture of open and closed chain exercise manoeuvres, a combination of concentric and eccentric exercise modalities and is focused on the overall strength of legs including the muscles around the hips and knee joints. An open kinetic chain is defined as “a combination of successively arranged joints in which the terminal segments can move freely” (60). Closed chain exercises are exercises or movements where the distal aspect of the extremity is fixed to an object that is stationary and proximal joints move (60). There are balance enhancement exercises as well. There are educational sessions integrated into the programme covering the basics of OA, its treatment, self-managing symptoms of OA and the benefits of maintaining a healthy lifestyle.

Control Group

The control group will continue with routine self-management or GP-management of knee OA which is offered in the community setting in the United Kingdom.

Follow up duration

Six weeks post-intervention.

The six week duration has been selected for this study based on a previous observational Quasi-Experiment study which reported six weeks of web-based treatment were effective to reduce knee pain (35).

Research Assessment

Outcome Measures in Rheumatology-Osteoarthritis Research Society International (OMERACT-OARSI) has recommended six domains as mandatory to be measured and reported in all hip and knee OA clinical trials (61). These are pain, physical function, quality of life, patient's global assessment of the

For peer review only - http://bmjopen.bmj.com/site/about/guidelines.xhtml

target joint, adverse events including mortality and Joint structure (in specific circumstances and depending on the intervention). The first four domains are measure by Numerical Rating Scale (NRS), Quantitate Sensory Testing (QST), Western Ontario and McMaster Universities Osteoarthritis Index (WOMAC), and the Arthritis Research UK Musculoskeletal Health Questionnaire (MSK-HQ). The adverse events will be reported at the end of the trial and joint structure will be reported based on x-rays and the findings of ultrasound scan of most painful knee. Time up and go test and 30-second sit to stand test are objective assessment of physical functions based on the recommendations of OARSI (62), thus the following outcome measures will be assessed:

1. Sleep
2. Numerical Rating Scale (NRS: Average Pain on the day of assessment on a scale of zero to ten)
3. Quantitate Sensory Testing – QST (Pressure pain threshold – PPT, Temporal summation – TS, and conditional pain modulation – CPM)
4. Inflammatory markers on ultrasound (Synovial fluid, synovial hypertrophy, and hypervascularity) (MSK-USS)
5. Muscle Thickness Assessment (MTA) of Vastus Lateralis Oblique (VLO) using MSK-USS
6. Maximum Voluntary Contraction (MVC)- isokinetic contraction of Quadriceps muscle
7. Biomarkers of insulin resistance
8. Physical functioning (Time up and go test, 30-second sit to stand test)
9. Western Ontario and McMaster Universities Osteoarthritis Index (WOMAC)
10. Pittsburgh Sleep Quality Index (PSQI)
11. General health questionnaire (MSK- HQ)
12. Body composition assessment by Bioimpedance Analysis (BIA)

Start and End dates

The trial has started in Feb 2019 and enrolment will end in December 2020.

Description of Intervention

Participants seeking care for OA will be informed of the study both orally and in writing. Those who qualify for the study will give signed informed consent, and the participants will be randomised to an intervention or control group. The consent will be taken by an experienced member of the research team. The exception applies to those participants who have not had a knee X-rays in the previous 12 months. These individuals will be called to the hospital, and after gaining valid consent, knee radiographs will be obtained, and their X-rays will be assessed by experienced staff. Once, a definite eligibility criterion (K/L score at least 1 or above) is established in such cases; the qualifying participants will be randomised to the intervention or control group.

Intervention group will have an assessment session with experienced staff and a Numerical Rating Scale (NRS) (63-65), the Western Ontario and McMaster Universities Osteoarthritis Index (WOMAC) (66, 67), the Arthritis Research UK Musculoskeletal Health Questionnaire (MSK-HQ)(68, 69), the Pittsburgh Sleep Quality Index (PSQI)(70-73), a 30-second sit to stand test (30CST)(74-76), a time to up and go test (TUG)(77, 78), the Quantitative Sensory Testing (QST)(79-85), Musculoskeletal ultrasound scan (MSK-USS)(86-90), a muscle Thickness Assessment (MTA) of vastus lateralis(91, 92), quadriceps muscle force generation during an isokinetic Maximum Voluntary Contraction (MVC)(93), urine and blood samples (94, 95) will be taken at baseline (refer to supplementary file for further information). These samples will be assessed for circulating levels of fasting Insulin, glucose, C-reactive protein (CRP), triglycerides, LDL, HDL, TNF-alpha, IL-6 and IL-1. Those who consent for

For peer review only - http://bmjopen.bmj.com/site/about/guidelines.xhtml

aspiration of synovial fluid will go through an ultrasound-guided aspiration (USGA) procedure (86-89). The intervention group will be given an actigraphy device (ActTrust). Their sleeping pattern will be recorded quantitatively.

The intervention group will then receive a link via email, which will be used to log-in to the Joint Academy online portal (see https://www.jointacademy.com). After log-in has been achieved, the exercise intervention will start. This will consist of a 6-week internet-based physical therapy programme that will provide information, exercise, contact details of a personal physiotherapist, education about lifestyle and behavioural changes. This intervention can be accessed using a smartphone, a tablet or a computer. The programme also encourages physical activity adherence by sending email prompts on a regular basis. Initially, participants will answer an online-questionnaire covering areas such as joint pain intensity, health-related quality of life, physical function as well as performing a physical test assessing lower limb strength. This questionnaire will form a part of the online baseline assessment. The exercise intervention will consist of knee and hip exercises along with some functional activities such as sit to stand and stairs climbing.

After the six week exercise intervention, participants will fill in the same questionnaire and perform the same physical tests, to enable evaluation. There are two face-to-face meetings between participants and physiotherapist/nurse, at enrolment and after six weeks. The physiotherapist will be available via asynchronous online chat or over the phone during the 6-week study period.

The control group will continue with their routine self-management which is offered in the community setup. They will be assessed on NRS, QST, WOMAC, MSK-HQ, PSQI, 30CST, TUG, MSK-USS, MTA of vastus lateralis, MVC of Quadriceps muscle, body composition, urine and blood samples at baseline. The control group will also use actigraphy to monitor sleep patterns. They will follow the routine management of knee OA recommended of the National Institute for Health and Care Excellence (NICE), which includes non-pharmacological and pharmacological management (96). They will be re-assessed after six weeks on the same outcome measures to determine if there has been an impact of self-management strategies.

Data Management

In the iBEAT-OA trial, data will be collected during the first and last session. Additional, weekly pain scores will be collected via an online portal and actigraphy will be used to collect sleep patterns. Online portal and actigraphy data will be collated by trained local research staff and data entry in a relational MS Access database will be completed in a standardised fashion. The clinical research forms (CRF) from the first and last study visit will be sent to the data entry site. A central data manager performs and monitors data entry. This will include questionnaires (MSK-HQ, PSQI, WOMAC) and data from QST, 30CST, TUG, MSK-USS, MTA of vastus lateralis, body composition and MVC of the Quadriceps muscle.

For peer review only - http://bmjopen.bmj.com/site/about/guidelines.xhtml

Statistical Analysis

Clinical trial data will be analysed using an intention-to-treat approach. We will compare outcome measures (e.g., pain sensitivity, pain scores, sleep patterns, and inflammatory measures) between the exercise and non-exercise groups controlling for baseline scores using appropriate parametric and non-parametric statistical tests. Additional observational secondary analyses (i.e., correlations between change in sleep patterns and change in pain measures) will be carried out using parametric statistics and adjusting for the relevant covariates. This missing data will be calculated based on multiple imputation. The SPSS package will be used for the statistical analyses.

Adverse Events

There are no serious adverse events reported with these exercises. These exercises have been trialled on seventy-five thousand patients from 2008 to 2017 with no serious adverse events (34, 59). There is a small chance of an increase in the knee, hip or back pain (24). We will monitor the pain levels of the patient on a weekly basis using an internet-based interface to monitor any increase in the knee, hip or low back pain. If the pain exacerbates to the level that the participant starts struggling with the activities of daily living, then they will be advised to stop participating in the study and will be advised to contact their GP.

Criteria for terminating the study

As the study involves only two assessments and does not involve investigational medicinal products or medical devices, and the same intervention has not given rise to any serious adverse events in over 70,000 participants in Sweden, it is not envisaged that circumstances will arise that require termination of this study.

Ethics and Dissemination

The study has received approval from the Research Ethics Committee (REC) (Ref: 18/EM/0154), Health Research Authority (HRA) (Protocol no: 18021) and the Nottingham University Hospitals NHS Trust Research & Innovation (R&I) department (Ref: 18RH004). Any modification to the approved protocol will require re-submission of modifications and further approval from the REC and the sponsor.

The study results will be submitted to Versus Arthritis, regulatory authorities, and a peer-reviewed journal for publication. Also, the results will be presented at national and international conferences. Study participants will also be informed of the results if requested.

Insurance and Indemnity

Insurance and indemnity for clinical study participants and study staff are covered within the NHS Indemnity Arrangements for clinical negligence claims in the NHS, issued under cover of HSG (96)48. There are no special compensation arrangements, but study participants may have recourse through the NHS complaints procedures.

The University of Nottingham as research sponsor indemnifies its staff, research participants and research protocols with both public liability insurance and clinical trials insurance. These policies include provision for indemnity in the event of a successful litigious claim for proven non-negligent harm.

For peer review only - http://bmjopen.bmj.com/site/about/guidelines.xhtml

Study Conduct

Study conduct will be subject to systems audit for inclusion of essential documents; permissions to conduct the study; CVs of study staff and training received; local document control procedures; consent procedures and recruitment logs; adherence to procedures defined in the protocol (e.g. inclusion / exclusion criteria, timeliness of visits); accountability of study materials and equipment calibration logs.

Study Data and Audits

Monitoring of study data shall include confirmation of informed consent; source data verification; data storage and data transfer procedures; local quality control checks and procedures, back-up and disaster recovery of any local databases and validation of data manipulation. This will be managed by the direct study team.

Entries on study forms will be verified by inspection against the source data. A sample of the forms (10%) will be checked on a regular basis for verification of all entries made. In addition, the subsequent capture of the data on the study database will be checked. Where corrections are required these will carry a full audit trail and justification.

Study data and evidence of monitoring and systems audits will be made available for inspection by the REC as required.

Record Retention and Archiving

In compliance with the ICH/GCP guidelines, regulations and in accordance with the University of Nottingham Code of Research Conduct and Research Ethics, the Chief or local Principal Investigator will maintain all records and documents regarding the conduct of the study. These will be retained for at least seven years or for longer if required. If the responsible investigator is no longer able to maintain the study records, a second person will be nominated to take over this responsibility.

The study master file held by the Chief Investigator on behalf of the Sponsor shall be finally archived at secure archive facilities at the University of Nottingham. This archive shall include all anonymised study databases and associated meta-data encryption codes.

Statement of Confidentiality

Individual participant medical or personal information obtained as a result of this study are considered confidential, and disclosure to third parties is prohibited. Participant confidentiality will be further ensured by utilising identification code numbers to correspond to treatment data in the computer files. Such medical information may be given to the participant's medical team and all appropriate medical personnel responsible for the participant's welfare.

Data generated as a result of this study will be available for inspection on request by the participating physicians, the University of Nottingham representatives, the REC, local R&D Departments and the regulatory authorities.

Data Sharing Statement

Data originating from the iBEAT-OA trial will be available upon request once the results from the trial have been published in a peer-reviewed publication. Researchers interested in accessing the data

For peer review only - http://bmjopen.bmj.com/site/about/guidelines.xhtml

will need to complete a "Data Access Proposal Form" and the investigators associated with iBEAT-OA will grant access to the data provided it is used research purposes only. No personal information of research participants will be shared as part of any data sharing.

Discussion

Knee OA remains one of the most common forms of OA and affects the majority of the population in the United Kingdom. Treatments include non-pharmacological and pharmacological management recommended by the National Institute for Health and Care Excellence (NICE). The non-pharmacological management recommends local muscles strengthening, general aerobic fitness, weight loss and using transcutaneous electrical nerve stimulation (TENS) as an adjunct to other forms of management (96). The majority of the population requires some guidance as to what exercises they should do and get referred to the Versus Arthritis website for basic exercises. If those exercises fail to make much difference or if patients struggle to understand these exercises, they are referred to a local physiotherapy department in the community. This means that some of these patients will have to travel to local community health centres or hospitals to see a physiotherapist and learn the relevant exercises.

The Joint Academy or other similar platform could work in-between the Versus Arthritis leaflet and referral to physiotherapy, thus cutting the cost of travelling, saving the time of the patient wasted in travelling and the time of physiotherapists so that they can treat other patients with more complex pathologies and needs.

Although iBEAT-OA will not specifically study the economic benefits of delivering the Joint Academy intervention instead of face to face physiotherapy, it will generate data on its efficacy which will serve as a starting point for any future health economic and cost-effectiveness analysis of such a method for relieving knee OA pain.

The majority of physiotherapists guide their patients on the type of exercises they should follow and review them a few times before the patients are recommended to self-manage OA and at this stage these patients get discharged. The compliance of patients afterward can decline as they may stop exercising. There are various reasons for this including adjusting lifestyle to include these exercises, pain during the exercises, lack of motivation and lack of professional monitoring (97-99). The Joint Academy platform is designed to keep these patients motivated by sending regular e-mails to remind them and by tracking their progress.

Social media is a powerful platform, which offers a connection between users and is a source of social interaction for a range of individuals. This can be used to promote health and to treat patients with OA (100-102). Joint Academy is aimed at using social media such as internet and digital application on a mobile phone to encourage patients with knee OA to self-manage their condition. This programme will educate and train them to stay 'in-control' of their knee OA which will improve their overall quality of life. This will lead to the overall psychological well-being of our population. This study will also encourage other researchers to study digital health platform, which will be the preferred way of communication and solution to health-related issues for next generations.

This study intends to establish the link between digital exercises, muscles strength, knee inflammation, sleep disturbance, pain and severity of knee OA. The intention is to check if exercising regularly can reduce pain, knee inflammation, sleep disturbance and slow down the progression of knee OA. If this

For peer review only - http://bmjopen.bmj.com/site/about/guidelines.xhtml

complicated link can be interpreted effectively, we may find a way to reduce the progression of knee OA.

Figure 1: A randomised controlled trial evaluating the efficacy of internet-Based Exercises Aimed at Treating Knee Osteoarthritis (iBEAT-OA) – PICOT Format

*NRS= Numerical Rating Scale, QST= Quantitative Sensory Testing, MSK USS= Musculoskeletal Ultrasound, MTA =Muscle Thickness Assessment, MVC= Maximum Voluntary Contraction- isokinetic contraction of Quadriceps muscle, TUG = Time up and go test, 30CST= 30-second sit to stand test, WOMAC= Western Ontario and McMaster Universities Osteoarthritis Index, PSQI= Pittsburgh Sleep Quality Index, MSK-HQ= Arthritis Research UK Musculoskeletal Health Questionnaire, BIA = Bioimpedance Assessment

Figure 2: iBEAT-OA Trial design. The flowchart summarises the design of iBEAT-OA trial.

Figure 3: Inclusion and Exclusion Criteria for iBEAT-OA trial

References

1. Peat G, McCarney R, Croft P. Knee pain and osteoarthritis in older adults: a review of community burden and current use of primary health care. Ann Rheum Dis. 2001;60(2):91-7.

For peer review only - http://bmjopen.bmj.com/site/about/guidelines.xhtml

2. Royal College of General P, McCormick A, Fleming D, Charlton J, Great B, Office of Population C, et al. Morbidity statistics from general practice : fourth national study 1991-1992. London: H.M.S.O.; 1995.
3. Conaghan PG, Dickson J, Grant RL. Care and management of osteoarthritis in adults: summary of NICE guidance. BMJ : British Medical Journal. 2008;336(7642):502-3.
4. Jevsevar DS. Treatment of osteoarthritis of the knee: evidence-based guideline, 2nd edition. The Journal of the American Academy of Orthopaedic Surgeons. 2013;21(9):571-6.
5. Pendleton A, Arden N, Dougados M, Doherty M, Bannwarth B, Bijlsma JW, et al. EULAR recommendations for the management of knee osteoarthritis: report of a task force of the Standing Committee for International Clinical Studies Including Therapeutic Trials (ESCISIT). Ann Rheum Dis. 2000;59(12):936-44.
6. Bennell KL, Hunter DJ, Hinman RS. Management of osteoarthritis of the knee. BMJ (Clinical research ed). 2012;345:e4934.
7. Hochberg MC, Altman RD, April KT, Benkhalti M, Guyatt G, McGowan J, et al. American College of Rheumatology 2012 recommendations for the use of nonpharmacologic and pharmacologic therapies in osteoarthritis of the hand, hip, and knee. Arthritis Care Res (Hoboken). 2012;64(4):465-74.
8. Bennell KL, Hinman RS. A review of the clinical evidence for exercise in osteoarthritis of the hip and knee. Journal of science and medicine in sport. 2011;14(1):4-9.
9. Messier SP, Loeser RF, Miller GD, Morgan TM, Rejeski WJ, Sevick MA, et al. Exercise and dietary weight loss in overweight and obese older adults with knee osteoarthritis: The arthritis, diet, and activity promotion trial. Arthritis & Rheumatism. 2004;50(5):1501-10.
10. Thomas KS, Muir KR, Doherty M, Jones AC, O'Reilly SC, Bassey EJ. Home based exercise programme for knee pain and knee osteoarthritis: randomised controlled trial. BMJ (Clinical research ed). 2002;325(7367):752.
11. van Baar ME, Assendelft WJ, Dekker J, Oostendorp RA, Bijlsma JW. Effectiveness of exercise therapy in patients with osteoarthritis of the hip or knee: a systematic review of randomized clinical trials. Arthritis and rheumatism. 1999;42(7):1361-9.
12. Chamberlain MA, Care G, Harfield B. Physiotherapy in osteoarthrosis of the knees. A controlled trial of hospital versus home exercises. International rehabilitation medicine. 1982;4(2):101-6.
13. Ettinger WH, Jr., Burns R, Messier SP, Applegate W, Rejeski WJ, Morgan T, et al. A randomized trial comparing aerobic exercise and resistance exercise with a health education program in older adults with knee osteoarthritis. The Fitness Arthritis and Seniors Trial (FAST). Jama. 1997;277(1):25-31.
14. Ettinger WH, Jr, Burns R, Messier SP, et al. A randomized trial comparing aerobic exercise and resistance exercise with a health education program in older adults with knee osteoarthritis: The fitness arthritis and seniors trial (fast). Jama. 1997;277(1):25-31.
15. Kovar PA, Allegrante JP, MacKenzie CR, Peterson MG, Gutin B, Charlson ME. Supervised fitness walking in patients with osteoarthritis of the knee. A randomized, controlled trial. Annals of internal medicine. 1992;116(7):529-34.
16. Minor MA, Hewett JE, Webel RR, Anderson SK, Kay DR. Efficacy of physical conditioning exercise in patients with rheumatoid arthritis and osteoarthritis. Arthritis and rheumatism. 1989;32(11):1396-405.
17. Fisher NM, Pendergast DR, Gresham GE, Calkins E. Muscle rehabilitation: its effect on muscular and functional performance of patients with knee osteoarthritis. Archives of physical medicine and rehabilitation. 1991;72(6):367-74.
18. Pisters MF, Veenhof C, van Meeteren NLU, Ostelo RW, de Bakker DH, Schellevis FG, et al. Long-Term effectiveness of exercise therapy in patients with osteoarthritis of the hip or knee: A systematic review. Arthritis Care & Research. 2007;57(7):1245-53.

For peer review only - http://bmjopen.bmj.com/site/about/guidelines.xhtml

19. Hurley MV, Walsh NE, Mitchell HL, Pimm TJ, Patel A, Williamson E, et al. Clinical effectiveness of a rehabilitation program integrating exercise, self-management, and active coping strategies for chronic knee pain: A cluster randomized trial. Arthritis Care & Research. 2007;57(7):1211-9.
20. Dunlop DD, Song J, Semanik PA, Sharma L, Chang RW. Physical activity levels and functional performance in the osteoarthritis initiative: a graded relationship. Arthritis and rheumatism. 2011;63(1):127-36.
21. Esser S, Bailey A. Effects of exercise and physical activity on knee osteoarthritis. Current pain and headache reports. 2011;15(6):423-30.
22. Lange AK, Vanwanseele B, Fiatarone Singh MA. Strength training for treatment of osteoarthritis of the knee: a systematic review. Arthritis and rheumatism. 2008;59(10):1488-94.
23. Mikesky AE, Mazzuca SA, Brandt KD, Perkins SM, Damush T, Lane KA. Effects of strength training on the incidence and progression of knee osteoarthritis. Arthritis Care & Research. 2006;55(5):690-9.
24. Fransen M, McConnell S, Harmer AR, Van der Esch M, Simic M, Bennell KL. Exercise for osteoarthritis of the knee: a Cochrane systematic review. British Journal of Sports Medicine. 2015;49(24):1554-7.
25. Li Y, Su Y, Chen S, Zhang Y, Zhang Z, Liu C, et al. The effects of resistance exercise in patients with knee osteoarthritis: a systematic review and meta-analysis. Clinical rehabilitation. 2016;30(10):947-59.
26. Baker K, McAlindon T. Exercise for knee osteoarthritis. Current opinion in rheumatology. 2000;12(5):456-63.
27. Raske Å, Norlin R. Injury Incidence and Prevalence among Elite Weight and Power Lifters. The American Journal of Sports Medicine. 2002;30(2):248-56.
28. Reeves RK, Laskowski ER, Smith J. Weight Training Injuries. The Physician and Sportsmedicine. 1998;26(2):67-96.
29. Reeves RK, Laskowski ER, Smith J. Weight Training Injuries. The Physician and Sportsmedicine. 1998;26(3):54-73.
30. Escamilla RF, Fleisig GS, Zheng N, Lander JE, Barrentine SW, Andrews JR, et al. Effects of technique variations on knee biomechanics during the squat and leg press. Medicine and science in sports and exercise. 2001;33(9):1552-66.
31. Peeler J, Ripat J. The effect of low-load exercise on joint pain, function, and activities of daily living in patients with knee osteoarthritis. The Knee. 2018.
32. UK AR. Exercises to manage Knee Pain [Available from: https://www.arthritisresearchuk.org/arthritis-information/exercises-to-manage-pain/knee-pain-exercises.aspx.
33. BOA. Better management of patients with OsteoArthritis (BOA) [Available from: https://boa.registercentrum.se/boa-in-english/better-management-of-patients-with-osteoarthritis-boa/p/By_o8GxVg.
34. Thorstensson CA, Garellick G, Rystedt H, Dahlberg LE. Better Management of Patients with Osteoarthritis: Development and Nationwide Implementation of an Evidence-Based Supported Osteoarthritis Self-Management Programme. Musculoskeletal care. 2015;13(2):67-75.
35. Nero H, Dahlberg J, Dahlberg EL. A 6-Week Web-Based Osteoarthritis Treatment Program: Observational Quasi-Experimental Study. J Med Internet Res. 2017;19(12):e422.
36. Skou ST, Roos EM. Good Life with osteoArthritis in Denmark (GLA:D™): evidence-based education and supervised neuromuscular exercise delivered by certified physiotherapists nationwide. BMC musculoskeletal disorders. 2017;18(1):72.
37. Talbot LA, Gaines JM, Huynh TN, Metter EJ. A Home-Based Pedometer-Driven Walking Program to Increase Physical Activity in Older Adults with Osteoarthritis of the Knee: A Preliminary Study. Journal of the American Geriatrics Society. 2003;51(3):387-92.

For peer review only - http://bmjopen.bmj.com/site/about/guidelines.xhtml

38. Lorig KR, Ritter PL, Laurent DD, Plant K. The internet-based arthritis self-management program: A one-year randomized trial for patients with arthritis or fibromyalgia. Arthritis Care & Research. 2008;59(7):1009-17.
39. Bossen D, Veenhof C, Van Beek KEC, Spreeuwenberg PMM, Dekker J, De Bakker DH. Effectiveness of a Web-Based Physical Activity Intervention in Patients With Knee and/or Hip Osteoarthritis: Randomized Controlled Trial. Journal of Medical Internet Research. 2013;15(11):e257.
40. Brooks MA, Beaulieu JE, Severson HH, Wille CM, Cooper D, Gau JM, et al. Web-based therapeutic exercise resource center as a treatment for knee osteoarthritis: a prospective cohort pilot study. BMC musculoskeletal disorders. 2014;15:158.
41. Dahlberg LE, Grahn D, Dahlberg JE, Thorstensson CA. A Web-Based Platform for Patients With Osteoarthritis of the Hip and Knee: A Pilot Study. JMIR research protocols. 2016;5(2):e115.
42. Allen KD, Arbeeva L, Callahan LF, Golightly YM, Goode AP, Heiderscheit BC, et al. Physical therapy vs internet-based exercise training for patients with knee osteoarthritis: results of a randomized controlled trial. Osteoarthritis and Cartilage. 2018;26(3):383-96.
43. Bennell KL, Nelligan R, Dobson F, Rini C, Keefe F, Kasza J, et al. Effectiveness of an Internet-Delivered Exercise and Pain-Coping Skills Training Intervention for Persons With Chronic Knee Pain: A Randomized TrialInternet-Delivered Exercise and Pain-Coping Skills Training for Knee Pain. Annals of internal medicine. 2017;166(7):453-62.
44. Wilcox S, Brenes GA, Levine D, Sevick MA, Shumaker SA, Craven T. Factors related to sleep disturbance in older adults experiencing knee pain or knee pain with radiographic evidence of knee osteoarthritis. J Am Geriatr Soc. 2000;48(10):1241-51.
45. Power JD, Perruccio AV, Badley EM. Pain as a mediator of sleep problems in arthritis and other chronic conditions. Arthritis Care & Research. 2005;53(6):911-9.
46. Smith MT, Quartana PJ, Okonkwo RM, Nasir A. Mechanisms by which sleep disturbance contributes to osteoarthritis pain: a conceptual model. Current pain and headache reports. 2009;13(6):447-54.
47. Leigh TJ, Hindmarch I, Bird HA, Wright V. Comparison of sleep in osteoarthritic patients and age and sex matched healthy controls. Ann Rheum Dis. 1988;47(1):40-2.
48. Doherty M, Smith J. Elusive 'alpha-delta' sleep in fibromyalgia and osteoarthritis. Ann Rheum Dis. 1993;52(3):245.
49. Harrison MM, Childs A, Carson PE. Incidence of undiagnosed sleep apnea in patients scheduled for elective total joint arthroplasty. The Journal of arthroplasty. 2003;18(8):1044-7.
50. Association ASD. International classification of sleep disorders. Revised: Diagnostic and coding manual. http://www absm org/PDF/ICSD pdf. 1997.
51. Campbell CM, Buenaver LF, Finan P, Bounds SC, Redding M, McCauley L, et al. Sleep, Pain Catastrophizing, and Central Sensitization in Knee Osteoarthritis Patients With and Without Insomnia. Arthritis Care Res (Hoboken). 2015;67(10):1387-96.
52. Gosset M, Berenbaum F, Salvat C, Sautet A, Pigenet A, Tahiri K, et al. Crucial role of visfatin/pre-B cell colony-enhancing factor in matrix degradation and prostaglandin E2 synthesis in chondrocytes: possible influence on osteoarthritis. Arthritis and rheumatism. 2008;58(5):1399-409.
53. Sowers M, Karvonen-Gutierrez CA, Palmieri-Smith R, Jacobson JA, Jiang Y, Ashton-Miller JA. Knee osteoarthritis in obese women with cardiometabolic clustering. Arthritis Care and Research. 2009;61(10):1328-36.
54. Puenpatom RA, Victor TW. Increased prevalence of metabolic syndrome in individuals with osteoarthritis: an analysis of NHANES III data. Postgraduate medicine. 2009;121(6):9-20.
55. Wang X, Hunter D, Xu J, Ding C. Metabolic triggered inflammation in osteoarthritis. Osteoarthritis and Cartilage. 2015;23(1):22-30.
56. Zhuo Q, Yang W, Chen J, Wang Y. Metabolic syndrome meets osteoarthritis. Nature Reviews Rheumatology. 2012;8:729.

For peer review only - http://bmjopen.bmj.com/site/about/guidelines.xhtml

57. Gregor MF, Hotamisligil GS. Inflammatory mechanisms in obesity. Annual review of immunology. 2011;29:415-45.
58. Fernandes GS, Sarmanova A, Warner S, Harvey H, Akin-Akinyosoye K, Richardson H, et al. Knee pain and related health in the community study (KPIC): a cohort study protocol. BMC musculoskeletal disorders. 2017;18(1):404-.
59. Academy J. Better management of patients with Osteoarthritis -BOA

2008 [Available from: https://boa.registercentrum.se/boa-in-english/better-management-of-patients-with-osteoarthritis-boa/p/By_o8GxVg.
60. GJ ETD. Closed Kinetic Chain Exercise: A Comprehensive Guide to Multiple Joint Exercise: Human Kinetics Publishers; 2001. 128 pages p.
61. Smith TO, Hawker GA, Hunter DJ, March LM, Boers M, Shea BJ, et al. The OMERACT-OARSI Core Domain Set for Measurement in Clinical Trials of Hip and/or Knee Osteoarthritis. J Rheumatol. 2019:jrheum.181194.
62. Dobson F, Hinman RS, Roos EM, Abbott JH, Stratford P, Davis AM, et al. OARSI recommended performance-based tests to assess physical function in people diagnosed with hip or knee osteoarthritis. Osteoarthritis and Cartilage. 2013;21(8):1042-52.
63. Ferreira-Valente MA, Pais-Ribeiro JL, Jensen MP. Validity of four pain intensity rating scales. PAIN®. 2011;152(10):2399-404.
64. Hjermstad MJ, Fayers PM, Haugen DF, Caraceni A, Hanks GW, Loge JH, et al. Studies Comparing Numerical Rating Scales, Verbal Rating Scales, and Visual Analogue Scales for Assessment of Pain Intensity in Adults: A Systematic Literature Review. Journal of Pain and Symptom Management. 2011;41(6):1073-93.
65. Karcioglu O, Topacoglu H, Dikme O, Dikme O. A systematic review of the pain scales in adults: Which to use? The American Journal of Emergency Medicine. 2018.
66. McConnell S, Kolopack P, Davis AM. The Western Ontario and McMaster Universities Osteoarthritis Index (WOMAC): a review of its utility and measurement properties. Arthritis Care & Research. 2001;45(5):453-61.
67. Bellamy N, Buchanan WW, Goldsmith CH, Campbell J, Stitt LW. Validation study of WOMAC: a health status instrument for measuring clinically important patient relevant outcomes to antirheumatic drug therapy in patients with osteoarthritis of the hip or knee. J Rheumatol. 1988;15(12):1833-40.
68. Hill JC, Kang S, Benedetto E, Myers H, Blackburn S, Smith S, et al. Development and initial cohort validation of the Arthritis Research UK Musculoskeletal Health Questionnaire (MSK-HQ) for use across musculoskeletal care pathways. BMJ Open. 2016;6(8).
69. Hill J, Kang S, Benedetto E, Myers H, Blackburn S, Smith S, et al. PMS74 - Development of the Musculoskeletal Health Questionnaire (MSK-HQ) for Use in Different Conditions and Different Healthcare Pathways. Value in Health. 2016;19(7):A544.
70. Backhaus J, Junghanns K, Broocks A, Riemann D, Hohagen F. Test–retest reliability and validity of the Pittsburgh Sleep Quality Index in primary insomnia. Journal of Psychosomatic Research. 2002;53(3):737-40.
71. Buysse DJ, Reynolds IIICF, Monk TH, Hoch CC, Yeager AL, Kupfer DJ. Quantification of Subjective Sleep Quality in Healthy Elderly Men and Women Using the Pittsburgh Sleep Quality Index (PSQI). Sleep. 1991;14(4):331-8.
72. Carpenter JS, Andrykowski MA. Psychometric evaluation of the pittsburgh sleep quality index. Journal of Psychosomatic Research. 1998;45(1):5-13.
73. Finan PH, Buenaver LF, Bounds SC, Hussain S, Park RJ, Haque UJ, et al. Discordance between pain and radiographic severity in knee osteoarthritis: Findings from quantitative sensory testing of central sensitization. Arthritis & Rheumatism. 2013;65(2):363-72.
74. Gill S, McBurney H. Reliability of performance-based measures in people awaiting joint replacement surgery of the hip or knee. Physiotherapy research international : the journal for researchers and clinicians in physical therapy. 2008;13(3):141-52.

For peer review only - http://bmjopen.bmj.com/site/about/guidelines.xhtml

75. Gill SD, de Morton NA, Mc Burney H. An investigation of the validity of six measures of physical function in people awaiting joint replacement surgery of the hip or knee. Clinical rehabilitation. 2012;26(10):945-51.
76. Jones CJ, Rikli RE, Beam WC. A 30-s chair-stand test as a measure of lower body strength in community-residing older adults. Research quarterly for exercise and sport. 1999;70(2):113-9.
77. Bennell K, Dobson F, Hinman R. Measures of physical performance assessments: Self-Paced Walk Test (SPWT), Stair Climb Test (SCT), Six-Minute Walk Test (6MWT), Chair Stand Test (CST), Timed Up & Go (TUG), Sock Test, Lift and Carry Test (LCT), and Car Task. Arthritis Care & Research. 2011;63(S11):S350-S70.
78. Podsiadlo D, Richardson S. The Timed "Up & Go": A Test of Basic Functional Mobility for Frail Elderly Persons. Journal of the American Geriatrics Society. 1991;39(2):142-8.
79. Arendt-Nielsen L, Nie H, Laursen MB, Laursen BS, Madeleine P, Simonsen OH, et al. Sensitization in patients with painful knee osteoarthritis. Pain. 2010;149(3):573-81.
80. Finan PH, Buenaver LF, Bounds SC, Hussain S, Park RJ, Haque UJ, et al. Discordance between pain and radiographic severity in knee osteoarthritis: findings from quantitative sensory testing of central sensitization. Arthritis and rheumatism. 2013;65(2):363-72.
81. Imamura M, Imamura ST, Kaziyama HH, Targino RA, Hsing WT, de Souza LP, et al. Impact of nervous system hyperalgesia on pain, disability, and quality of life in patients with knee osteoarthritis: a controlled analysis. Arthritis and rheumatism. 2008;59(10):1424-31.
82. Lee YC, Lu B, Bathon JM, Haythornthwaite JA, Smith MT, Page GG, et al. Pain sensitivity and pain reactivity in osteoarthritis. Arthritis Care Res (Hoboken). 2011;63(3):320-7.
83. Neogi T, Frey-Law L, Scholz J, Niu J, Arendt-Nielsen L, Woolf C, et al. Sensitivity and sensitisation in relation to pain severity in knee osteoarthritis: trait or state? Ann Rheum Dis. 2015;74(4):682-8.
84. Neogi T, Guermazi A, Roemer F, Nevitt MC, Scholz J, Arendt-Nielsen L, et al. Association of Joint Inflammation With Pain Sensitization in Knee Osteoarthritis: The Multicenter Osteoarthritis Study. Arthritis & rheumatology (Hoboken, NJ). 2016;68(3):654-61.
85. Wessel J. The reliability and validity of pain threshold measurements in osteoarthritis of the knee. Scand J Rheumatol. 1995;24(4):238-42.
86. D'Agostino MA, Conaghan P, Le Bars M, Baron G, Grassi W, Martin-Mola E, et al. EULAR report on the use of ultrasonography in painful knee osteoarthritis. Part 1: Prevalence of inflammation in osteoarthritis. Annals of the Rheumatic Diseases. 2005;64(12):1703-9.
87. Hayashi D, Roemer FW, Katur A, Felson DT, Yang S-O, Alomran F, et al. Imaging of Synovitis in Osteoarthritis: Current Status and Outlook. Seminars in Arthritis and Rheumatism. 2011;41(2):116-30.
88. Karim Z, Wakefield RJ, Quinn M, Conaghan PG, Brown AK, Veale DJ, et al. Validation and reproducibility of ultrasonography in the detection of synovitis in the knee: a comparison with arthroscopy and clinical examination. Arthritis and rheumatism. 2004;50(2):387-94.
89. Sarmanova A, Hall M, Fernandes GS, Bhattacharya A, Valdes AM, Walsh DA, et al. Association between ultrasound-detected synovitis and knee pain: a population-based case-control study with both cross-sectional and follow-up data. Arthritis research & therapy. 2017;19(1):281.
90. Sarmanova A, Hall M, Moses J, Doherty M, Zhang W. Synovial changes detected by ultrasound in people with knee osteoarthritis – a meta-analysis of observational studies. Osteoarthritis and Cartilage. 2016;24(8):1376-83.
91. Franchi MV, Longo S, Mallinson J, Quinlan JI, Taylor T, Greenhaff PL, et al. Muscle thickness correlates to muscle cross-sectional area in the assessment of strength training-induced hypertrophy. Scandinavian journal of medicine & science in sports. 2017.
92. Strasser EM, Draskovits T, Praschak M, Quittan M, Graf A. Association between ultrasound measurements of muscle thickness, pennation angle, echogenicity and skeletal muscle strength in the elderly. AGE. 2013;35(6):2377-88.

For peer review only - http://bmjopen.bmj.com/site/about/guidelines.xhtml

93. Tan J, Balci N, Sepici V, Gener FA. Isokinetic and isometric strength in osteoarthrosis of the knee. A comparative study with healthy women. Am J Phys Med Rehabil. 1995;74(5):364-9.
94. Berry PA, Jones SW, Cicuttini FM, Wluka AE, Maciewicz RA. Temporal relationship between serum adipokines, biomarkers of bone and cartilage turnover, and cartilage volume loss in a population with clinical knee osteoarthritis. Arthritis & Rheumatism. 2011;63(3):700-7.
95. Richette P, Poitou C, Garnero P, Vicaut E, Bouillot J-L, Lacorte J-M, et al. Benefits of massive weight loss on symptoms, systemic inflammation and cartilage turnover in obese patients with knee osteoarthritis. Annals of the Rheumatic Diseases. 2011;70(1):139-44.
96. Excellence NIfHaC. Osteoarthritis: care and management 2014 [Available from: https://www.nice.org.uk/guidance/cg177/chapter/1-Recommendations#non-pharmacological-management-2

97. Campbell R, Evans M, Tucker M, Quilty B, Dieppe P, Donovan JL. Why don't patients do their exercises? Understanding non-compliance with physiotherapy in patients with osteoarthritis of the knee. Journal of Epidemiology and Community Health. 2001;55(2):132-8.
98. Jack K, McLean SM, Moffett JK, Gardiner E. Barriers to treatment adherence in physiotherapy outpatient clinics: A systematic review. Manual Therapy. 2010;15(3):220-8.
99. Marks R, Allegrante JP. Chronic osteoarthritis and adherence to exercise: a review of the literature. Journal of aging and physical activity. 2005;13(4):434-60.
100. Eytan T, Benabio J, Golla V, Parikh R, Stein S. Social Media and the Health System. The Permanente Journal. 2011;15(1):71-4.
101. Moorhead SA, Hazlett DE, Harrison L, Carroll JK, Irwin A, Hoving C. A New Dimension of Health Care: Systematic Review of the Uses, Benefits, and Limitations of Social Media for Health Communication. Journal of Medical Internet Research. 2013;15(4):e85.
102. Thackeray R, Crookston BT, West JH. Correlates of Health-Related Social Media Use Among Adults. Journal of Medical Internet Research. 2013;15(1):e21.

For peer review only

Authors' contributions:

Sameer Gohir is the primary author and all other authors are secondary. Dr Ana M. Valdes is the main supervisor and leading this project. Dr Paul Greenhaff and Dr Abhishek Abhishek are secondary supervisors. All authors have equally contributed to this article.

Acknowledgement:

We would like to thank Dr Michella Hall (Assistant Professor, Division of Physiotherapy and Rehabilitation Sciences, The University of Nottingham) and Tony Kelly (Research Metrologist, Academic Rheumatology, University of Nottingham) for their valuable contribution into designing, monitoring and implementing this study.

For peer review only - http://bmjopen.bmj.com/site/about/guidelines.xhtml

Funding statement:

This study is part-funded by the Arthritis Research UK Pain Centre (University of Nottingham) and part-funded by Nottingham Biomedical Research Centre (BRC).

Trial Sponsor:

Nottingham University

Head of Research Governance, Research and Innovation,

University of Nottingham,

East Atrium

Jubilee Conference Centre

Triumph Road

Nottingham

NG8 1DH

Competing interests statement:

None Declared

For peer review only

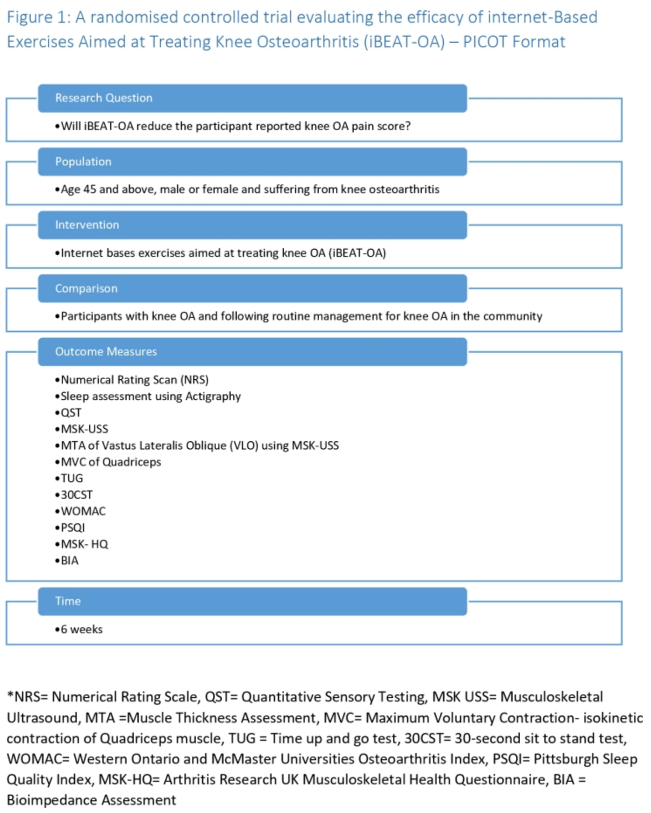

Figure 1: A randomised controlled trial evaluating the efficacy of internet-Based Exercises Aimed at Treating Knee Osteoarthritis (iBEAT-OA) – PICOT Format

140x198mm (300 x 300 DPI)

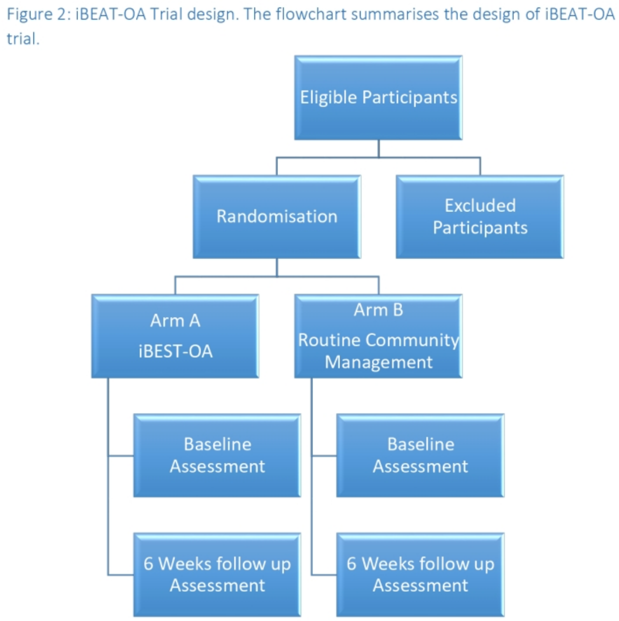

Figure 2: iBEAT-OA Trial design. The flowchart summarises the design of iBEAT-OA trial.

140x198mm (300 x 300 DPI)

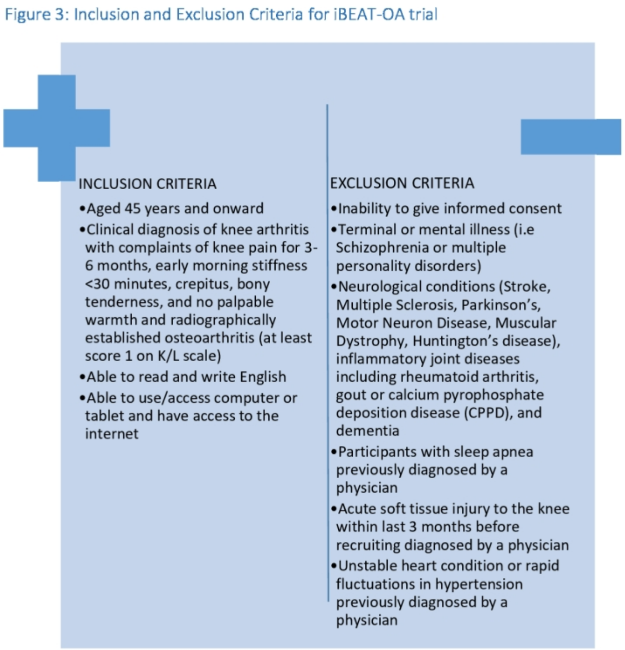

Figure 3: Inclusion and Exclusion Criteria for iBEAT-OA trial

140x198mm (300 x 300 DPI)

Time up and Go: The participant will start in a seated position.
The participant will stand up upon therapist's command, walk 3 meters, turn around, walk back to the chair and sits down.
The time will stop when the participant is seated.
The subject can use an assistive device. If the assistive device is used, it will be documented.
NOTE: A practice trial will be completed before the timed trial

30 seconds sit to stand. The 30-Second Chair Test is administered using a folding chair without arms, with a seat height of 17 inches (43.2 cm). The chair, with rubber tips on the legs, is placed against a wall to prevent it from moving.
The participant is seated in the middle of the chair, back straight; feet approximately shoulder width apart and placed on the floor at an angle slightly back from the knees, with one foot slightly in front of the other to help maintain balance. Arms are crossed at the wrists and held against the chest.
Demonstrate the task both slowly and quickly.
Have the participant practice a repetition or 2 before completing the test.
If a participant must use their arms to complete the test, they are scored 0.
The participant is encouraged to complete as many full stands as possible within 30 seconds.
The participant is instructed to fully sit between each stand.
While monitoring the participant's performance to ensure proper form, the tester silently counts the completion of each correct stand. The score is the total number of stands within 30 seconds (more than halfway up at the end of 30 seconds counts as a full stand). Incorrectly executed stands are not counted.
The 30-second chair stand involves recording the number of stands a person can complete in 30 seconds rather than the amount of time it takes to complete a pre-determined number of repetitions.

Muscle strength Assessment: Isokinetic testing will be done at 60 and 180 degrees of flexion and the participant will be in sitting position with hips and knees strapped to keep the position standardised. Isokinetic torque will be measured in the seated position on a Computer Sports Medicine, Inc (CSMI) HUMAC / NORM Testing and Rehabilitation System (Model 770) isokinetic dynamometer at 60 and 180° s^{-1} angular velocities. There will be 30 seconds rest between each testing and one-minute break between 60 and 180° s^{-1} angular velocities.

Ultrasound and Muscle Thickness Assessment (MTA) of Vastus Lateralis Oblique (VLO): An ultrasound will be used to image both knee joints using a Toshiba Aplio SSA-770A machine with a multi-frequency (7 – 12 Hz). This equipment belongs to the university and is CE marked. During the ultrasound scan, the maximal synovial thickness and effusion depth will be measured in millimetres using the longitudinal axis. Suprapatellar pouch, medial and lateral recess of the knees will be assessed for synovial thickening, synovial fluid/effusion and for positive power Doppler.

Maximal muscle thickness will be measured in transversal images as the distance between the superficial and the deep fascia at the widest distance and scan will be conducted at the midpoint between greater trochanter and knee joint. The pennation angle is defined as the angle between muscle fibres and the deep fascia of the muscle. Pennation angles will be measured in the longitudinal ultrasound image for three fibres of vastus medialis, and the average of these three measurements will be used for further analysis.

An ultrasonic probe will be used to direct ultrasonic waves onto the knee joint during sonography, and a computer converts the signals received so that they can be presented on the screen. There is no radiation exposure to ultrasound due to the lack of radioactive rays and no detrimental side effects.

Pressure Pain threshold (PPT)

PPT is a non-invasive test during which the sensitivities of the nerves are assessed by recording the smallest force applied to the skin. When this force by the surface area of the skin is applied (pressure), this will be felt as mild, temporary pain and recorded. The pressure probe used consists of a rod with an end the size of a 5p piece, mounted in a handheld device connected to a computer. The force with which the probe is pressed onto the skin is gradually increased until the participant indicates (by pressing a button) that the sensation has changed from pressure to pain. The probe is then automatically immediately taken off the skin. The probe will be used on knee (medial joint line, supero-lateral, supero-medial and tibialis anterior) using a standardised protocol used in other studies within the Pain Centre. The participants will be familiarised with the test before it is administered so that they know what to expect and how to respond.

Temporal Summation (TS)

The mechanical temporal summation is a non-invasive test during which repetitive mechanical stimulation is applied over a short period to get their augmented response. Increased pain response to a repeated mechanical stimulus may indicate enhanced central sensitisation. The test site will be a suprapatellar region (5 cm proximal from the central part of patella).

A 256mN weighted pinprick stimulator will be used and applied perpendicular to the skin of suprapatellar region of affected knee (5cm proximal from the centre of patella). The participant will be asked to rate the pain or sharpness they experience from 0-10 where 0 indicates no pain or sharpness and 10 indicates the most intense pain or sharpness imaginable. Numerical Rating Scale (NRS) with verbal descriptors will be used. The response of the participant will be recorded. The same stimulator at the same site will be applied ten times repeatedly at a rate of 1/second. The size of the site would be kept approximately 1 cm square. At the end of the series of 10 pinpricks, the participant will be asked to rate the pain or sharpness which they experience averaged over the whole series of 10 stimuli using the same NRS. The mechanical temporal summation reading will be calculated as the difference between two ratings which is second rating minus the first rating.

Continuous Pain Modulation (CPM)

CPM will be done along with PPT testing. The reference point for PPT testing will tibialis anterior on the most painful knee. Verbal instructions will be given to the participant. The Numerical Rating Scale (NRS) target will be ≥4 out of 10 from the cuff pressure. Staff/ clinician will wrap 7.5cm wide tourniquet cuff around the contralateral arm to the knee being tested. The lower rim of tourniquet cuff will be kept 3cm proximal to cubital fossa. Systolic pressure will be set to 20mmHg higher than systolic blood pressure of the participant. After target pressure is achieved, the participant will be asked to rate sensation in the arm from 0-10. The participant will be asked to make hand grips until NRS of 4 will be reached. NRS rating will be asked every five hand grips.

Once NRS of 4 will be achieved, the probe of algometer will be applied in the same manner as before to tibialis anterior site (during PPT testing). Once the participant presses the button, the probe will be withdrawn, and cuff will be released from the elbow. Participants will be advised to wait until cuff evoked pain subsides before re-test, and a minimum of 1 minute should be spared. PPT test will be repeated again (without the cuff now). Their difference in PPT score (with conditioning – without conditioning) will establish the CPM effects. Positive value predicts efficient and negative value predicts in-effective CPM.

For peer review only

Sites for QST

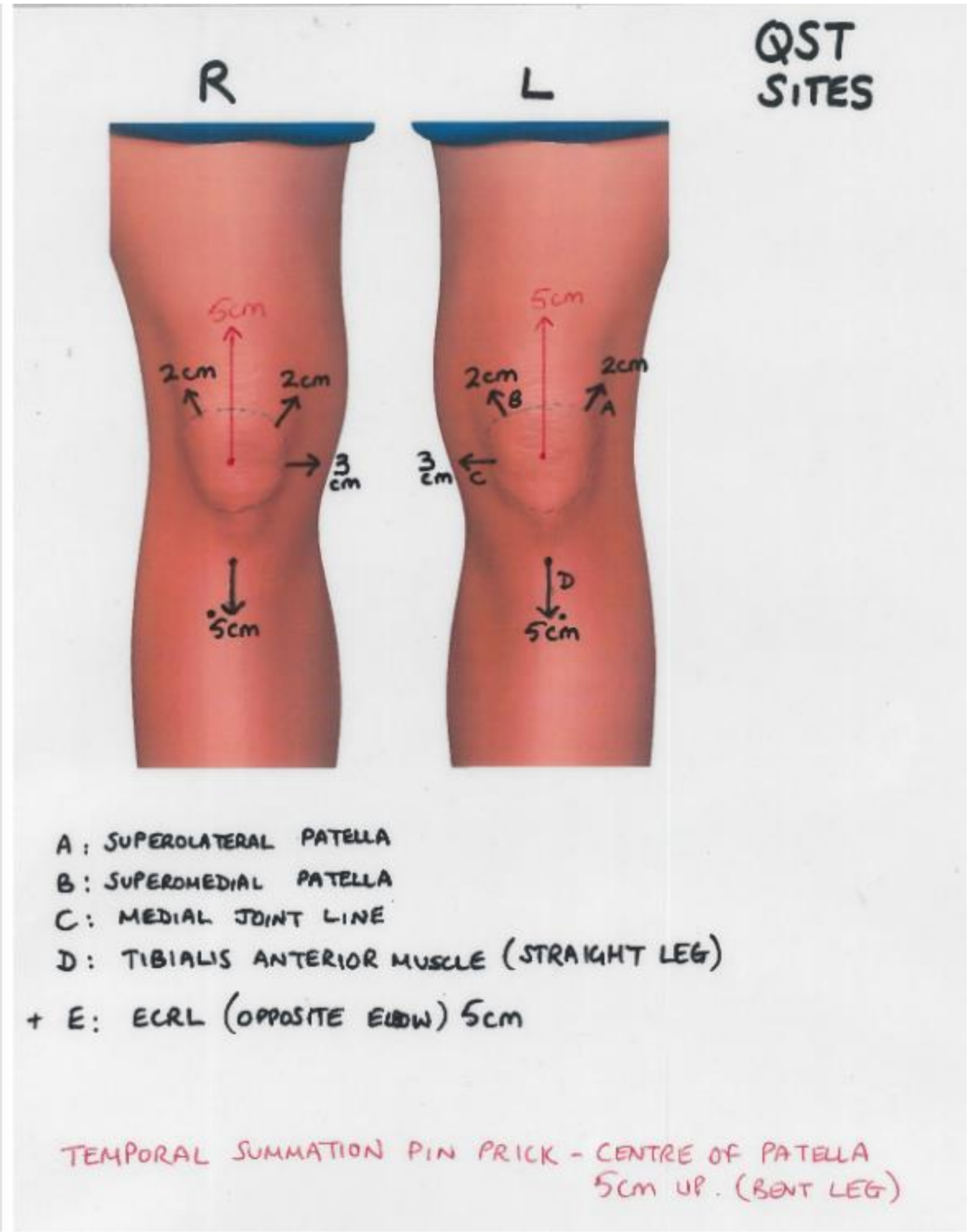

Biomarkers:
Insulin, glucose, C- reactive protein (CRP), triglycerides, LDL, HDL, TNF-alpha, IL6,

SPIRIT

STANDARD PROTOCOL ITEMS: RECOMMENDATIONS FOR INTERVENTIONAL TRIALS

SPIRIT 2013 Checklist: Recommended items to address in a clinical trial protocol and related documents*

Section/item	Item No	Description
**Administrative information**		
Title	1	Descriptive title identifying the study design, population, interventions, and, if applicable, trial acronym – **ON P1**
Trial registration	2a	Trial identifier and registry name. If not yet registered, name of intended registry – **ON P1**
	2b	All items from the World Health Organization Trial Registration Data Set
Protocol version	3	Date and version identifier – **added now- Ref to footer section**
Funding	4	Sources and types of financial, material, and other support **– ON P18**
Roles and responsibilities	5a	Names, affiliations, and roles of protocol contributors**- ON P1**
	5b	Name and contact information for the trial sponsor**- ON P18**
	5c	Role of study sponsor and funders, if any, in study design; collection, management, analysis, and interpretation of data; writing of the report; and the decision to submit the report for publication, including whether they will have ultimate authority over any of these activities**- ON P18**
	5d	Composition, roles, and responsibilities of the coordinating centre, steering committee, endpoint adjudication committee, data management team, and other individuals or groups overseeing the trial, if applicable (see Item 21a for data monitoring committee)- **NA**
**Introduction**		
Background and rationale	6a	Description of research question and justification for undertaking the trial, including summary of relevant studies (published and unpublished) examining benefits and harms for each intervention –**P2-4**
	6b	Explanation for choice of comparators **P2-4**
Objectives	7	Specific objectives or hypotheses **P4**

For peer review only - http://bmjopen.bmj.com/site/about/guidelines.xhtml

Trial design	8	Description of trial design including type of trial (eg, parallel group, crossover, factorial, single group), allocation ratio, and framework (eg, superiority, equivalence, noninferiority, exploratory)- **P4**

Methods: Participants, interventions, and outcomes

Study setting	9	Description of study settings (eg, community clinic, academic hospital) and list of countries where data will be collected. Reference to where list of study sites can be obtained- **P4**
Eligibility criteria	10	Inclusion and exclusion criteria for participants. If applicable, eligibility criteria for study centres and individuals who will perform the interventions (eg, surgeons, psychotherapists) – **Figure 3**
Interventions	11a	Interventions for each group with sufficient detail to allow replication, including how and when they will be administered **– P6-9 and supplementary document 'Assessment Explained'**
	11b	Criteria for discontinuing or modifying allocated interventions for a given trial participant (eg, drug dose change in response to harms, participant request, or improving/worsening disease) – **P8**
	11c	Strategies to improve adherence to intervention protocols, and any procedures for monitoring adherence (eg, drug tablet return, laboratory tests)- **P7- 3rd Paragraph under Description of Intervention**
	11d	Relevant concomitant care and interventions that are permitted or prohibited during the trial- **NA**
Outcomes	12	Primary, secondary, and other outcomes, including the specific measurement variable (eg, systolic blood pressure), analysis metric (eg, change from baseline, final value, time to event), method of aggregation (eg, median, proportion), and time point for each outcome. Explanation of the clinical relevance of chosen efficacy and harm outcomes is strongly recommended **– P6-7**
Participant timeline	13	Time schedule of enrolment, interventions (including any run-ins and washouts), assessments, and visits for participants. A schematic diagram is highly recommended (see Figure)- **Figure 2**
Sample size	14	Estimated number of participants needed to achieve study objectives and how it was determined, including clinical and statistical assumptions supporting any sample size calculations- **P5- Sample size and justification**
Recruitment	15	Strategies for achieving adequate participant enrolment to reach target sample size – **P5**- Refer to Randomisation and blinding section

Methods: Assignment of interventions (for controlled trials)

Allocation:

Sequence generation	16a	Method of generating the allocation sequence (eg, computer-generated random numbers), and list of any factors for stratification. To reduce predictability of a random sequence, details of any planned restriction (eg, blocking) should be provided in a separate document that is unavailable to those who enrol participants or assign interventions- **P5**
Allocation concealment mechanism	16b	Mechanism of implementing the allocation sequence (eg, central telephone; sequentially numbered, opaque, sealed envelopes), describing any steps to conceal the sequence until interventions are assigned- **P5**
Implementation	16c	Who will generate the allocation sequence, who will enrol participants, and who will assign participants to interventions- **P5**
Blinding (masking)	17a	Who will be blinded after assignment to interventions (eg, trial participants, care providers, outcome assessors, data analysts), and how- **P5**
	17b	If blinded, circumstances under which unblinding is permissible, and procedure for revealing a participant's allocated intervention during the trial- **P5**

Methods: Data collection, management, and analysis

Data collection methods	18a	Plans for assessment and collection of outcome, baseline, and other trial data, including any related processes to promote data quality (eg, duplicate measurements, training of assessors) and a description of study instruments (eg, questionnaires, laboratory tests) along with their reliability and validity, if known. Reference to where data collection forms can be found, if not in the protocol – **(The references to the questionnaires used are cited in the literature section of the manuscript -p12-17)**
	18b	Plans to promote participant retention and complete follow-up, including list of any outcome data to be collected for participants who discontinue or deviate from intervention protocols- **P7- 3rd Paragraph under Description of Intervention**
Data management	19	Plans for data entry, coding, security, and storage, including any related processes to promote data quality (eg, double data entry; range checks for data values). Reference to where details of data management procedures can be found, if not in the protocol **P7- Data management**
Statistical methods	20a	Statistical methods for analysing primary and secondary outcomes. Reference to where other details of the statistical analysis plan can be found, if not in the protocol – **P8- Statistical Analysis section**

For peer review only - http://bmjopen.bmj.com/site/about/guidelines.xhtml

	20b	Methods for any additional analyses (eg, subgroup and adjusted analyses) - **P8- Statistical Analysis section**
	20c	Definition of analysis population relating to protocol non-adherence (eg, as randomised analysis), and any statistical methods to handle missing data (eg, multiple imputation) **P8- Statistical Analysis section**
**Methods: Monitoring**		
Data monitoring	21a	Composition of data monitoring committee (DMC); summary of its role and reporting structure; statement of whether it is independent from the sponsor and competing interests; and reference to where further details about its charter can be found, if not in the protocol. Alternatively, an explanation of why a DMC is not needed **- The sponsor is not involved in data analysis, as there are no commercial products involved there are no competing interests.**
	21b	Description of any interim analyses and stopping guidelines, including who will have access to these interim results and make the final decision to terminate the trial. **Given the safety profile of the intervention, no stopping of the study is foreseen.**
Harms	22	Plans for collecting, assessing, reporting, and managing solicited and spontaneously reported adverse events and other unintended effects of trial interventions or trial conduct- **P8- Adverse Event section**
Auditing	23	Frequency and procedures for auditing trial conduct, if any, and whether the process will be independent from investigators and the sponsor- **P9- Study data and audits section**
**Ethics and dissemination**		
Research ethics approval	24	Plans for seeking research ethics committee/institutional review board (REC/IRB) approval – **P8- Ethical and Dissemination section**
Protocol amendments	25	Plans for communicating important protocol modifications (eg, changes to eligibility criteria, outcomes, analyses) to relevant parties (eg, investigators, REC/IRBs, trial participants, trial registries, journals, regulators) **P8- Ethical and Dissemination section**
Consent or assent	26a	Who will obtain informed consent or assent from potential trial participants or authorised surrogates, and how (see Item 32) **P7- First Paragraph under Description of Intervention section**
	26b	Additional consent provisions for collection and use of participant data and biological specimens in ancillary studies, if applicable **P7- Second Paragraph re: Synovial fluid aspiration**

For peer review only - http://bmjopen.bmj.com/site/about/guidelines.xhtml

Confidentiality	27	How personal information about potential and enrolled participants will be collected, shared, and maintained in order to protect confidentiality before, during, and after the trial- **P8- Record Retention and confidentiality section**
Declaration of interests	28	Financial and other competing interests for principal investigators for the overall trial and each study site **– P19 and 20**
Access to data	29	Statement of who will have access to the final trial dataset, and disclosure of contractual agreements that limit such access for investigators **P9- Record Retention and confidentiality section**
Ancillary and post-trial care	30	Provisions, if any, for ancillary and post-trial care, and for compensation to those who suffer harm from trial participation- **P9- Insurance section**
Dissemination policy	31a	Plans for investigators and sponsor to communicate trial results to participants, healthcare professionals, the public, and other relevant groups (eg, via publication, reporting in results databases, or other data sharing arrangements), including any publication restrictions- **P8 Ethics and Dissemination**
	31b	Authorship eligibility guidelines and any intended use of professional writers **– No professional writers will be engaged and only researchers who meet the ICMJE authorship criteria (**http://www.icmje.org/recommendations/browse/roles-and-responsibilities/defining-the-role-of-authors-and-contributors.html) will be included as co-authors
	31c	Plans, if any, for granting public access to the full protocol, participant-level dataset, and statistical code **– Individual anonymised data and statistical code will be made available upon request to legitimate researchers after the manuscript reporting the RCT findings are published**
**Appendices**		
Informed consent materials	32	Model consent form and other related documentation given to participants and authorised surrogates **– Check additional documents**
Biological specimens	33	Plans for collection, laboratory evaluation, and storage of biological specimens for genetic or molecular analysis in the current trial and for future use in ancillary studies, if applicable **- P7- Description of intervention**

*It is strongly recommended that this checklist be read in conjunction with the SPIRIT 2013 Explanation & Elaboration for important clarification on the items. Amendments to the protocol should be tracked and dated. The SPIRIT checklist is copyrighted by the SPIRIT Group under the Creative Commons "Attribution-NonCommercial-NoDerivs 3.0 Unported" license.

For peer review only - http://bmjopen.bmj.com/site/about/guidelines.xhtml

TIDieR
Template for Intervention Description and Replication

The TIDieR (Template for Intervention Description and Replication) Checklist*: IBEAT-OA RCT

Information to include when describing an intervention and the location of the information

Item number	Item	Where located ** Primary paper (page or appendix number)	Other † (details)
	**BRIEF NAME**		
**1.**	Provide the name or a phrase that describes the intervention.	P1	______________
	**WHY**		
**2.**	Describe any rationale, theory, or goal of the elements essential to the intervention.	P2-3	______________
	**WHAT**		
**3.**	Materials: Describe any physical or informational materials used in the intervention, including those provided to participants or used in intervention delivery or in training of intervention providers. Provide information on where the materials can be accessed (e.g. online appendix, URL).		Ref to consent form and PIS under supplementary documentation
**4.**	Procedures: Describe each of the procedures, activities, and/or processes used in the intervention, including any enabling or support activities.		Ref to 'Assessments explained' document uploaded
	**WHO PROVIDED**		
**5.**	For each category of intervention provider (e.g. psychologist, nursing assistant), describe their expertise, background and any specific training given.	Sameer Gohir (Physio): Bio-impedance	______________

For peer review only - http://bmjopen.bmj.com/site/about/guidelines.xhtml

		analysis, ultrasound scan, muscle strength testing<br>Tony Kelly (Research Nurse): Bloods, Faecal & urine samples, QST, TUAG & 30 sec sit to stand tests	
	**HOW**		
**6.**	Describe the modes of delivery (e.g. face-to-face or by some other mechanism, such as internet or telephone) of the intervention and whether it was provided individually or in a group.	Assessments and re-assessments- face to face, intervention online as per P7- description of events	______________
	**WHERE**		
**7.**	Describe the type(s) of location(s) where the intervention occurred, including any necessary infrastructure or relevant features.	P4- Trial Design	______________
	**WHEN and HOW MUCH**		

For peer review only

**8.**	Describe the number of times the intervention was delivered and over what period of time including the number of sessions, their schedule, and their duration, intensity or dose.	P7- Description of events	______________
	**TAILORING**		
**9.**	If the intervention was planned to be personalised, titrated or adapted, then describe what, why, when, and how.	NA	______________
	**MODIFICATIONS**		
**10.‡**	If the intervention was modified during the course of the study, describe the changes (what, why, when, and how).	NA	______________
	**HOW WELL**		
**11.**	Planned: If intervention adherence or fidelity was assessed, describe how and by whom, and if any strategies were used to maintain or improve fidelity, describe them.	NA	______________
**12.‡**	Actual: If intervention adherence or fidelity was assessed, describe the extent to which the intervention was delivered as planned.	NA	______________

** **Authors** - use N/A if an item is not applicable for the intervention being described. **Reviewers** – use '?' if information about the element is not reported/not sufficiently reported.

† If the information is not provided in the primary paper, give details of where this information is available. This may include locations such as a published protocol or other published papers (provide citation details) or a website (provide the URL).
‡ If completing the TIDieR checklist for a protocol, these items are not relevant to the protocol and cannot be described until the study is complete.

* We strongly recommend using this checklist in conjunction with the TIDieR guide (see *BMJ* 2014;348:g1687) which contains an explanation and elaboration for each item.

* The focus of TIDieR is on reporting details of the intervention elements (and where relevant, comparison elements) of a study. Other elements and methodological features of studies are covered by other reporting statements and checklists and have not been duplicated as part of the TIDieR checklist. When a **randomised trial** is being reported, the TIDieR checklist should be used in conjunction with the CONSORT statement (see www.consort-statement.org) as an extension of **Item 5 of the CONSORT 2010 Statement.** When a **clinical trial protocol** is being reported, the TIDieR checklist should be used in conjunction with the SPIRIT statement as an extension of **Item 11 of the SPIRIT 2013 Statement** (see www.spirit-statement.org). For alternate study designs, TIDieR can be used in conjunction with the appropriate checklist for that study design (see www.equator-network.org).

For peer review only - http://bmjopen.bmj.com/site/about/guidelines.xhtml
